# Interfacial Failure and Self‐Healing in Solid‐State Batteries

**DOI:** 10.1002/adma.74666

**Published:** 2026-08-13

**Authors:** Xinxin Zhu, Tengfei Dai, Wendi Dou, Zhiheng Li, Kaihua Wen, Yanqun Lv, Ming Zhao, Jun Lu, Zaiping Guo

**Affiliations:** ^1^ Department of Materials Science and Engineering City University of Hong Kong Hong Kong China; ^2^ College of Chemical and Biological Engineering Zhejiang University Hangzhou China

**Keywords:** electro‐chemo‐mechanical failure, interface, interfacial self‐healing, solid‐state battery, solid‐state electrolyte

## Abstract

Solid‐state batteries hold great promise for simultaneously improving energy density and intrinsic safety. However, their practical application is severely impeded by interfacial instabilities arising from coupled mechanical, chemical, and electrochemical degradation during cycling, ultimately resulting in rapid performance decay. Although conventional strategies, such as interfacial coatings or electrode structural optimization, have partially improved electrochemical performance, their inherently static nature renders them ill‐equipped to adapt to the continuous and dynamic evolution of interfacial damage during long‐term operation. To address these persistent challenges, dynamic interfacial self‐healing has emerged as a compelling strategy for developing highly durable and stable solid‐state batteries. In this review, we first discuss the origins and evolution of mechanical, chemical, and electrochemical failures at the interface, highlighting their intricate interplay. Recent progress in self‐healing strategies, including physical flow, chemical restoration, external stimuli, and electric fields was subsequently analyzed to solve specific interfacial failure behavior. Perspectives on the emerging strategies and key challenges for achieving high self‐healing efficiency were provided in the end. The development of self‐healing mechanisms presents a highly viable route toward the realization of robust, low‐pressure solid‐state batteries.

## Graphical abstract

Dynamic interfacial self‐healing offers an adaptive route to mitigate coupled mechanical, chemical, and electrochemical degradation in solid‐state batteries. This review connects evolving interfacial failure mechanisms with physical‐flow, chemical‐restoration, stimuli‐responsive, and electric‐field‐assisted repair strategies, highlighting targeted self‐healing design for durable and low‐pressure solid‐state battery systems.

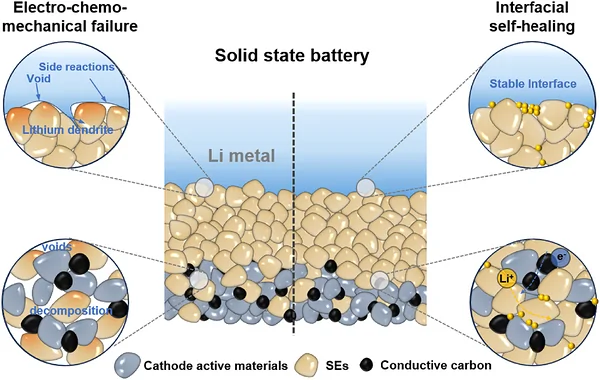

## 1 Introduction

Driven by the rapid growth of the electric vehicle market and the urgent demand for high‐energy‐density and safe energy storage, solid‐state batteries (SSBs) have emerged as a promising next‐generation technology [1, 2, 3]. By replacing flammable liquid electrolytes with thermally stable solid‐state electrolytes (SSEs), SSBs offer the potential to achieve higher energy density while fundamentally improving safety [4, 5, 6]. Despite recent reports of SSBs with energy densities above 400 Wh kg^−^^1^, their cycle life remains insufficient for practical application, especially for inorganic SSEs that often rely on high stack pressure to maintain interfacial contact [7, 8, 9]. This limitation fundamentally arises from the rigidity of the SSEs and the restricted solid‐solid point contact mode at the interface. In contrast to liquid systems, where ubiquitous wetting maintains continuous ion transport, SSBs depend on rigid interfaces formed among SSEs, conductive carbon, and active particles to facilitate electrochemical reactions [10, 11, 12]. Inherent mismatches in the volume, structure, and mechanical properties of these components cause heterogeneous charge transport and strongly coupled electro‐chemo‐mechanical degradation (Figure 1) [13, 14]. Specifically, anisotropic lattice strain within the cathode induces localized stress, leading to intergranular cracking and delamination. The anode/SSE interface is plagued by severe side reactions and dendrite growth, which continuously degrade physical contact [15, 16, 17]. Furthermore, the narrow electrochemical stability window of most SSEs makes it exceedingly difficult to stabilize both electrode interfaces simultaneously [18, 19]. The structural or electrochemical failure of any single component cascades, severely limiting the lifespan of the entire battery.

**FIGURE 1:**
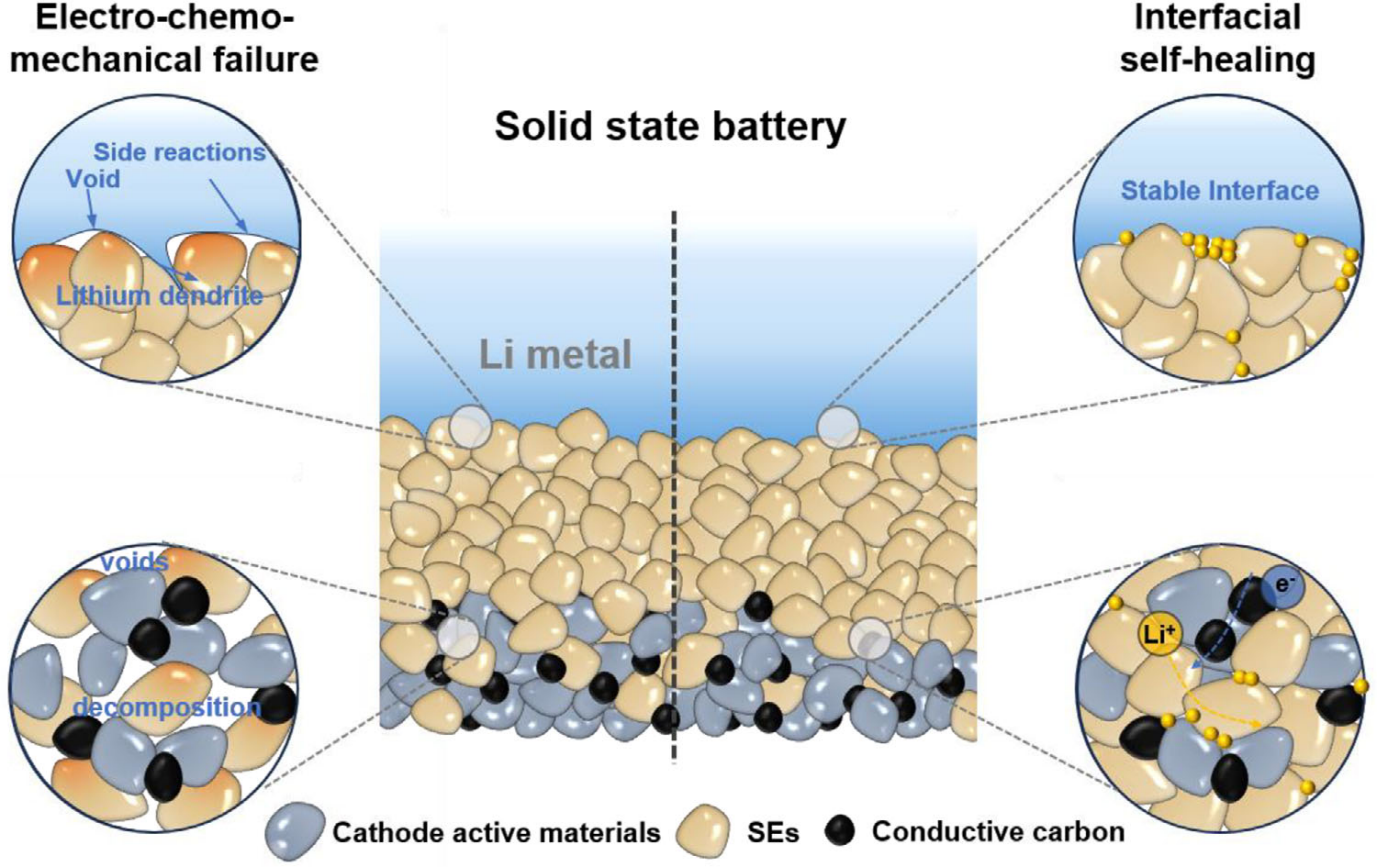
Schematic diagram of interfacial failure and self‐healing in SSBs.

Understanding the failure behaviors of key battery materials and their interfaces is essential for developing highly stable SSBs. In recent years, many excellent reviews have summarized interfacial failure mechanisms and discussed corresponding interface engineering strategies [20, 21, 22, 23, 24]. However, most of them mainly focus on degradation behaviors in specific battery systems and emphasize static protection approaches, such as surface coatings, artificial interlayers, and structural optimization, to delay performance decay. With the deepening of interfacial degradation, increasing work shows that even modified electrodes and predesigned protective interfaces inevitably undergo continuously evolving failure during long‐term cycling [25, 26, 27]. This indicates that static and passive protection strategies alone are insufficient to adapt to the dynamic evolution of solid‐solid interfaces during battery operation. Therefore, it is important to further shift the design concept from passive protection to active self‐healing, maintaining the long‐term interfacial stability under continuous cycling. In contrast to conventional static designs, self‐healing can dynamically respond to the damage generated during battery operation [28, 29, 30]. When microcracks, local debonding, or early‐stage interfacial failures emerge, the healing process is activated to restore interfacial integrity and sustain both mechanical and electrochemical continuity [31, 32]. A variety of self‐healing approaches have been reported, including flow‐mediated gap filling, dynamic covalent bond reconstruction, and thermal/pressure/electric‐field responsive repair, offering insights into extending the cycle life of high‐capacity SSBs [33, 34, 35, 36, 37]. Interfacial self‐healing not only promotes in situ crack closure and stress redistribution but also regulates local interfacial chemistry, thereby alleviating electro‐chemo‐mechanical degradation (Figure 1). Based on this consideration, a comprehensive review framework is needed to connect mechanical and (electro)chemical degradation with dynamic self‐healing strategies from the perspective of the entire solid‐state battery system. In this way, dynamic self‐healing is not treated as an isolated functional material design, but as a unifying framework that links failure mechanisms, interfacial evolution, and repair strategies, guiding the development of active and sustainable interface stabilization strategies.

In this review, we focus on the interfaces of SSB, systematically analyzing their failure mechanisms from mechanical, chemical, and electrochemical perspectives with a particular emphasis on their intricate coupled behaviors. Building upon this mechanistic understanding, we then discuss tailored self‐healing strategies to address specific failure modes at representative cathode and anode interfaces. These strategies are classified into physical flow, chemical restoration, and stimuli‐responsive mechanisms. To finish, we propose forward‐looking design principles to balance self‐repairing efficiency with overall electrochemical performance. By elucidating how dynamic self‐healing mitigates interfacial failure, this review provides critical insights for designing robust interfaces, ultimately guiding the development of long‐life, safe, and reliable SSBs.

## 2 Electro‐Chemo‐Mechanical Failure Mechanisms in SSBs

In SSBs, the interface serves as the primary pathway for charge transport. Thus, its long‐term stability directly determines the cyclic life and performance [38, 39, 40]. Unlike liquid electrolytes, SSEs lack the natural wetting properties and fluidity, making interfacial evolution during electrochemical cycling exceptionally complex [41, 42]. Driven by the highly coupled processes of mechanical instability and (electro)‐chemical degradation, the interface gradually fails, leading to a rapid decline in battery performance [43, 44, 45]. Therefore, a deep understanding of these degradation pathways is an essential prerequisite for engineering stable interfaces and achieving high‐performance SSBs. This section systematically discusses the mechanical, chemical, and electrochemical failure mechanisms of typical cathode and anode materials, and elucidates their intrinsic correlations, providing a holistic view of the interfacial evolution.

### 2.1 Mechanical Stress‐Induced Physical Degradation

Due to the inherently rigid interfaces in SSBs, mechanical degradation is a prevalent and critical cause of interfacial failure [46, 47]. This degradation is primarily driven by the volumetric fluctuations of active materials during Li (de)intercalation, where long‐term cycling ultimately results in severe interfacial contact loss [48, 49]. Mechanical failure mechanisms differ among active materials, as they are intricately tied to their respective charge‐discharge mechanisms. This section focuses on the mechanical degradation mechanisms of representative electrodes, including layered oxides, conversion‐type materials, Li metal, and silicon anodes, offering crucial theoretical insights for mitigating interfacial physical contact failures.

#### 2.1.1 Anisotropic Lattice Strain and Volume Change in Cathodes

Among all cathode materials, layered oxides are widely regarded as one of the most promising active materials for SSBs [50, 51, 52]. In a typical composite cathode, the architecture mainly consists of active materials and conductive carbon in polymer‐based SSBs, whereas inorganic‐based systems also include SSEs to ensure ionic percolation [11, 53, 54]. In these configurations, all interfaces are solid‐solid contacts. During the battery assembly process, the microscopic gaps and voids are almost unavoidable due to the intrinsic rigidity of the materials and their surface irregularities, severely hindering ion and electron transport [48, 55]. Such poor solid‐solid contact usually requires external large stack pressure to establish sufficient interfacial contact. Applying higher pressure generally improves physical contact between the active materials and the conductors, reducing overall porosity, while also making the material more brittle [7, 9, 56]. Both active materials and SSEs have their own mechanical tolerance limits. When the stack pressure reaches critical pressure, the components undergo irreversible mechanical damage and structural failure, leading to poor performance. Even one component damage within the composite cathode can trigger the whole battery's performance degradation.

Electrochemical cycling further exacerbates these structural challenges. Mechanical degradation in cathodes primarily stems from the repeated Li insertion and extraction within the active materials. The intrinsic lattice evolution of the active material acts as the fundamental driver of interfacial instability [57, 58]. Take high‐nickel layered oxides as an example, during delithiation (Figure 2a), they fundamentally evolve from the initial hexagonal phase (H1) to a monoclinic phase (M), followed by a second hexagonal phase (H2), and ultimately a third hexagonal phase (H3) [59, 60]. The H1‐M‐H2 transitions are accompanied by a gradual expansion along the c‐axis due to the increasing electrostatic repulsion between adjacent oxygen layers [61]. However, during the phase transition from H2 to H3, as more Ni^3+^ is oxidized to Ni^4+^, the Ni–O covalency increases, reducing the effective negative charge of the oxygen ions and leading to an abrupt collapse of the c‐axis [62, 63, 64]. Furthermore, these volume changes increase with increased nickel content in the layered material, generating immense internal mechanical stress [65, 66]. The resulting stress also depends strongly on particle morphology and size. While polycrystalline secondary particles are highly susceptible to severe intergranular cracking because of the random orientation of primary crystallites, single‐crystalline particles provide better structural integrity by eliminating internal grain boundaries [67, 68, 69]. However, they still suffer from concentrated surface stress. When the stress is transmitted to SSEs, most inorganic SSEs cannot accommodate the accumulated stress because of their high Young's modulus (Figure 2b) [70, 71, 72]. As cycling proceeds, microcracks initiated inside particles propagate across the boundaries of the cathode active material and further extend into the brittle SSE framework [73, 74]. Constrained by their intrinsically low fracture toughness (0.23 MPa·m^1/2^ for Li_6_PS_5_Cl), the SSEs inevitably fracture and sever the ion‐conducting pathways [75, 76]. This mechanical failure is cumulative. As cycling continues, crack propagation across different phases builds up a complex three‐dimensional fracture network within the composite electrode (Figure 2c), posing a serious threat to the cycling life and overall stability of the battery [77]. While external stack pressure is expected to maintain tight physical contact, it would intensify local stress concentration and accelerate fatigue of the electrode components.

**FIGURE 2:**
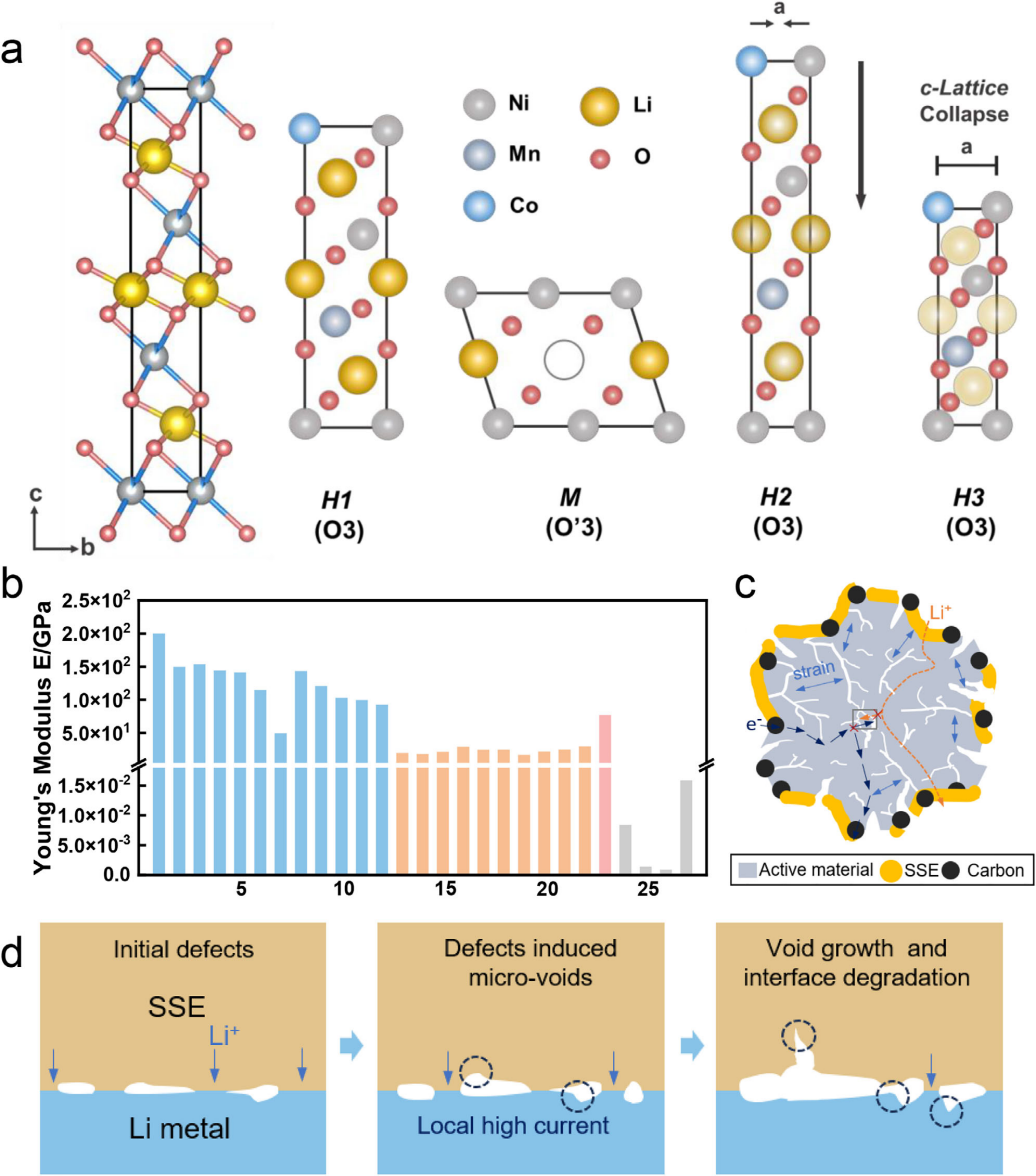
(a) Schematic diagram of the crystal structure of high‐nickel layered oxide. (b) The Young's modulus of the SSEs. (1‐Li_0.33_La_0.57_TiO_3_, 2‐Li_7_La_3_Zr_2_O_12_, 3‐Li_6.5_La_3_Zr_1.5_Ta_0.5_O_12_, 4‐Li_5_La_3_Ta_2_O_12_, 5‐Li_5_La_3_Nb_2_O_12_, 6‐Li_1.3_Al_0.3_Ti_1.7_(PO_4_)_3_, 7‐Li_1.5_Al_0.5_Ge_1.5_(PO_4_)_3_, 8‐LiTi_2_(PO_4_)_3_, 9‐NaZr_2_(PO_4_)_3_, 10‐Li_3_PO_4_, 11‐Li_3_OCl, 12‐Li_3_OBr, 13‐80Li_2_S‐20P_2_S_5_ glass, 14‐75Li_2_S‐25P_2_S_5_, 15‐Li_10_GeP_2_S_12_, 16‐Li_10_SnP_2_S_12_, 17‐Li_10_SiP_2_S_12_, 18‐Li_3_PS_4_, 19‐Li_7_P_3_S_11_, 20‐Li_6_PS_5_Cl, 21‐Li_6_PS_5_Br, 22‐Li_6_PS_5_I, 23‐LiPON, 24‐PEO/LiTFSI, 25‐PEO/LiClO_4_, 26‐PVDF/PEG/LiBF_4_, 27‐PAN/LiClO_4_) (c) Schematic diagram of cathode mechanical failure. (d) Schematic diagram of defect‐induced Li metal mechanical failure.

Beyond layered oxides, conversion‐type cathodes such as sulfur are popular for SSBs due to their exceptionally high theoretical specific capacity of 1675 mAh g^−1^ [78]. However, sulfur cathodes suffer from more severe volume change during cycling than layered oxides [49]. Despite differences in the conversion mechanism between liquid and solid, Li_2_S is verified as the final discharge product in both [79, 80]. This full S‐to‐Li_2_S conversion is accompanied by a huge theoretical volume expansion of up to about 80%, imposing severe mechanical stress on the surrounding SSE framework [81, 82]. This large strain mismatch not only causes local cracking, but also undermines the conductive carbon network and ionic transport pathways throughout the composite cathode. The resulting electrical and ionic isolation of the active material, commonly referred to as “dead sulfur”, markedly increases interfacial resistance and causes rapid capacity decay [83].

#### 2.1.2 Stripping‐Induced Voids and Stress Heterogeneity in Anodes

Li metal is considered a “grail” anode benefiting from its ultrahigh theoretical specific capacity and the lowest electrochemical potential [84, 85]. Its low melting point (180.5°C) and low hardness (0.6 on the Mohs scale) give it excellent plastic deformability, allowing it to accommodate interfacial stress through dislocation glide and creep flow [86, 87]. However, growing evidence indicates that Li metal anodes undergo a fatigue process during electrochemical cycling that resembles that of structural metals [88, 89]. This process is characterized by the formation, growth, and accumulation of micro‐voids, which can eventually lead to short‐circuit failure. Notably, such degradation can occur even at current densities below the critical current density. In the initial stage, failure is mainly governed by defect‐induced fatigue arising from poor solid‐solid contact. SSE surfaces are typically rough and contain defects such as grain boundaries and voids, so contact with Li metal is often limited to discrete point‐contact regions (Figure 2d) [90]. This problem is particularly severe in oxide electrolytes such as Li_7_La_3_Zr_2_O_12_, where the surface component of Li_2_CO_3_ further reduces wettability and leads to highly nonuniform interfacial contact [91]. As a result, the electric field and current density become locally concentrated at these contact points, creating preferred sites for Li deposition. At the same time, stress concentration develops around these defects and further enlarges existing gaps [92]. Unlike liquid electrolytes, which can refill gaps through capillary action, rigid SSEs cannot close the physical spaces left by metal recession in real time, leading to a continuous increase in interfacial resistance.

Li stripping behavior plays a central role in interfacial mechanical degradation. During stripping, Li^+^ is removed from the metal surface. To maintain intimate interfacial contact, atomic vacancies generated at the interface must be rapidly eliminated. This process mainly relies on two pathways: self‐diffusion of Li atoms toward the interface and creep‐mediated replenishment, as reported by Chen et al. [93]. In their study, Li metal was confined within nanoscale mixed ionic‐electronic conductor (MIEC) tubules, where Li could reversibly deposit and strip along the tubular structure. They showed that the shape change of Li during cycling was not dominated by bulk translation or dislocation‐mediated flow, but mainly proceeded through interfacial‐diffusional Coble creep along the MIEC/Li phase boundary. During Li stripping, a void plug could form between the residual Li and the solid electrolyte side. However, Li extraction still continued through the MIEC wall or surface, indicating that Li atoms were transported through diffusion‐mediated pathways rather than by simple mechanical displacement. The kinetic mismatch between Li‐vacancy diffusion and the creep response of the metal is regarded as the fundamental origin of mechanical failure, as evidenced by operando imaging. As current density increases, the rate of Li extraction can far exceed the rates of atomic diffusion and creep‐mediated replenishment. This kinetic lag causes vacancy supersaturation at contact points, which eventually evolves into macroscopic voids [14]. As cycling proceeds, microscopic voids migrate laterally along the SSE interface under local chemical‐potential gradients associated with curvature differences at void edges. Isolated nanovoids gradually merge into larger physical gaps. This morphological evolution sharply reduces the effective contact area, forcing external stack pressure onto remaining contact points and causing local stress at these sites to rise dramatically [94]. In situ monitoring with embedded optical fiber sensors shows that such a highly heterogeneous stress distribution disrupts electrochemical equilibrium and induces strong local electric‐field concentration [95]. This amplified nonuniformity not only accelerates further local failure, but also creates the physical space required for dendrite nucleation during subsequent plating, leading to battery short circuit.

Si‐based anodes, another high anode material, including nano‐Si, micron‐sized Si, and Si/C composites also undergo severe and repeated volume fluctuation during cycling [96, 97]. During lithiation, Si expands up to 300% in volume theoretically, generating enormous mechanical stress within the geometrically confined space of SSB [98]. This mechanical burden is especially severe for micron‐sized Si, whose larger particle size and lower fracture tolerance make it more vulnerable to rapid pulverization [99, 100, 101]. In contrast, nanostructured Si generally provides better structural stability because of its reduced dimensions. Si/C composites further mitigate these challenges through a resilient carbon matrix that acts as a mechanical buffer, accommodating volume expansion and preserving a stable conductive network [102, 103]. Despite reduced Si content and structural optimizations, modeling by Liu et al. suggests that repeated expansion and contraction of Si particles still generate a large mechanical driving force that threatens the structural integrity of the surrounding SSE framework [104]. Therefore, whether through stripping‐induced void formation in Li metal anodes or the large volume strain of Si‐based anodes, the strong coupling between interfacial cavitation and repeated volume fluctuation defines the stress‐driven origin of eventual mechanical failure at the anode side.

### 2.2 (Electro)Chemical Failure at Interfaces

While mechanical stress physically disrupts interfacial contact, the thermodynamic and electrochemical mismatches between electrodes and SSEs dictate the intrinsic (electro)chemical failure. Most SSEs exhibit narrow electrochemical stability windows and profound thermodynamic instability when in direct contact with highly reactive electrodes (such as Li metal anodes or high‐voltage cathodes) [105, 106]. Consequently, spontaneous chemical reactions occur even at rest, while voltage‐driven electrochemical decomposition accelerates during cycling. These parasitic processes lead to the continuous formation of highly resistive ion‐blocking interphases and the irreversible depletion of active Li. This section systematically discusses the chemical and electrochemical coupling degradation mechanisms at both the cathode and anode interfaces.

#### 2.2.1 (Electro)Chemical Incompatibility and Interphase Evolution in Cathodes

Beyond mechanical degradation, the intrinsic chemical potential mismatch between cathode materials and SSEs drives the formation of a space charge layer (SCL) [107, 108]. Upon contact, this mismatch forces transient Li redistribution to achieve equilibrium, creating a Li‐depleted region on the SSE side. While initially an electrostatic effect, the SCL actively alters the local chemical environment and triggers deeper interfacial degradation. For instance, Cui et al. demonstrate that this redistribution significantly increases interfacial resistance during the initial charging stage by in situ electrochemical impedance spectroscopy [109]. The formation and dynamic evolution of the SCL are illustrated in Figure 3a–d. Driven by a relatively higher Li chemical potential (µ_Li_), both Li^+^ and electrons migrate from the sulfide SSE to the cathode until equilibrium is reached (*µ*_Li_ = $\tilde{\mu }$_Li+_ + $\tilde{\mu }$_e−_). This spontaneous migration results in a Li^+^‐depletion layer in the SSE and a corresponding Li^+^‐enrichment region in the cathode (Figure 3b) [110]. Since Li^+^ is the sole charge carrier in the SSE, this depletion drastically lowers local ionic conductivity and induces high interfacial resistance. During the initial charging stage, Li^+^ in the SSE migrates toward the anode. However, Li^+^ de‐intercalation from the cathode does not occur immediately, as it requires the electrode potential to overcome a specific energy barrier. Consequently, the Li^+^‐depletion layer on the SSE side thickens, and the enriched region on the cathode side widens (Figure 3c). In this phase, the voltage profile exhibits a capacitor‐like oxidation slope (Figure 3a). Once the electrode potential exceeds the redox plateau of the cathode, Li^+^ finally de‐intercalates and migrates to the SSE to compensate for the depletion layer (Figure 3d). Despite this compensation, the SCL persists throughout subsequent cycling, acting as a continuous kinetic barrier for Li^+^ transport. Furthermore, during long‐term battery aging, the SCL dynamically evolves and becomes structurally more complex due to ongoing interfacial side reactions and ion exchange [111].

**FIGURE 3:**
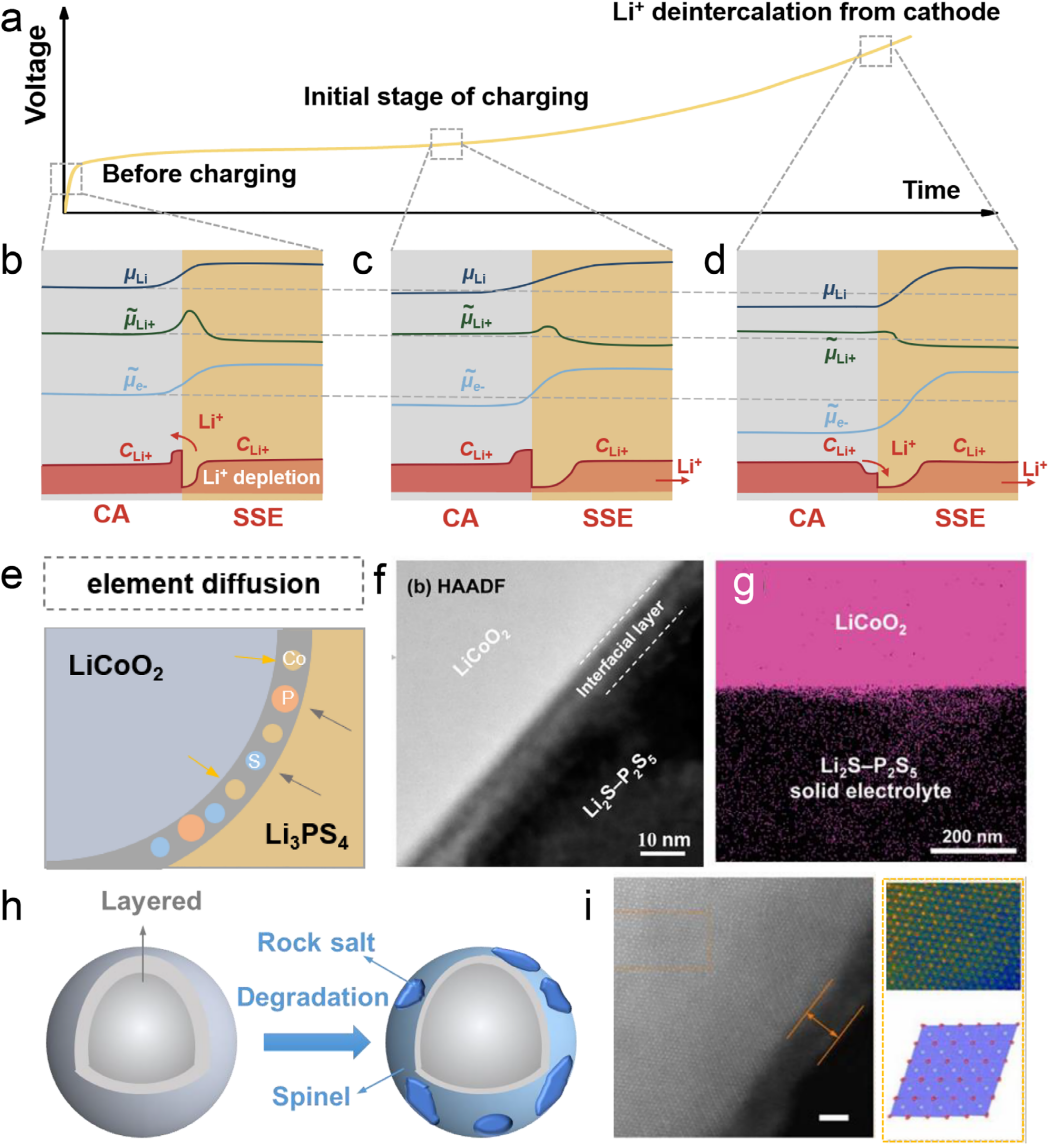
(a–d) Schematic illustrations of the formation and evolution of the SCL during the charging process. The SCL effect on the battery charging voltage profile is presented in (a), and (b–d) illustrate the distribution of the Li chemical potential *µ*_Li_, Li^+^ electrochemical potential $\tilde{\mu }$_Li+_, Li^+^ concentration *c*_Li+_, and the e^–^ electrochemical potential $\tilde{\mu }$_e−_. Adapted with permission [110]. Copyright 2023, Elsevier. (e) The element diffusion phenomenon occurs between the LiCoO_2_ electrode and the Li_3_PS_4_ SSE. (f) High‐angle annular dark field (HAADF) TEM images of the LiCoO_2_/Li_2_S‐P_2_S_5_ interface after initial charging. Reproduced with permission [112]. Copyright 2010, American Chemical Society. (h) Interface degradation of layered oxide cathodes after cycling. (i) Cross‐sectional HAADF‐STEM EDX mapping for the Co element near the LiCoO_2_ / Li_2_S‐P_2_S_5_ SSE interface after initial charging. Reproduced with permission [116]. Copyright 2020, Springer Nature.

The intrinsic chemical potential mismatch between cathode materials and SSEs also drives element interdiffusion and interfacial reactions. The resulting insulating decomposition products, such as metal sulfides, phosphides, and oxidized species are formed and gradually accumulate at the interface, increasing interfacial resistance and severely compromising the long‐term stability of SSBs. As shown in Figure 3e, Sakuda et al. directly visualized the interphase formed between LiCoO_2_ and Li_2_S‐P_2_S_5_ electrolytes by transmission electron microscopy (TEM), while energy‐dispersive x‐ray spectroscopy (EDS) confirmed mutual interdiffusion of Co, P, and S across the interface (Figure 3f,g) [112]. First‐principles calculations by Gao et al. further revealed that, at the LiCoO_2_/*β*‐Li_3_PS_4_ interface, interfacial phosphorus preferentially bonds with surface oxygen rather than sulfur, leading to the formation of PO_3_S tetrahedra [113]. Continuous degradation of the interface will result in the loss of active Li, leading to capacity reduction. Interfacial chemical degradation highly depends on both thermodynamics and reaction kinetics. Koerver et al. revealed that degradation remained limited at the early stage and only became obvious after prolonged contact for about 16 h, despite a strong thermodynamic driving force [114]. Thus, chemical degradation at solid‐solid interfaces is a kinetically controlled multistep process, in which activation barriers determine the reaction pathway and rate.

Electrochemical processes significantly accelerate the instability of the cathode/SSE interface, especially under high operating voltages. During this process, both the bulk cathode active materials and SSEs undergo structural degradation, accompanied by parasitic side reactions at their interface. First, most SSEs, particularly sulfide and polymer‐based systems, possess relatively narrow oxidation stability windows, often falling below the charging voltage of high‐Ni cathodes (typically around 4.3 V vs. Li/Li^+^) [115]. As a result, the structure of the electrolyte severely degrades, leading to a pronounced decrease in ionic conductivity. For cathode active materials, at high states of delithiation, Li extraction from layered oxide cathodes destabilizes lattice oxygen, leading to oxygen vacancy formation and cation mixing. This process often induces irreversible surface transformation from the layered phase to spinel‐like or rock‐salt phases, resulting in loss of active Li‐storage sites and release of reactive oxygen species (Figure 3h,i) [116]. Consequently, the highly reactive Ni^4+^ and released oxygen oxidize the surrounding sulfide SSE into resistive SO_x_^–^ and PO_x_^–^ containing species. Concurrently, the cathode surface is reduced to lower‐valence Ni species, forming a NiO‐like rock‐salt reconstruction layer [114]. These reactions yield a dense, highly resistive interphase that severely blocks Li^+^ transport across the boundary. In situ x‐ray diffraction detects the formation of cathode‐electrolyte interphases and surface reconstruction layers as early as the end of the first charge. As cycling proceeds, the interphase continuously accumulates, hindering delithiation and causing gradual performance decay. However, this degradation tends to approach saturation after about 50 cycles, when the resistive layer nearly covers the entire cathode surface [117]. The resistive interphase impacts the charging process greatly, because the increased charge‐transfer resistance hinders smooth Li extraction and causes pronounced polarization and capacity loss. Although halide SSEs such as Li_3_InCl_6_ show better compatibility with high‐voltage cathodes due to their higher oxidation resistance, slow electrochemical degradation is also observed during long‐term cycling at halide‐based SSE/active material interfaces. The main chemical failure mechanism between them is proven to be highly dependent on the state of charge (SOC) [118]. SSE decomposition tends to dominate aging at low SOCs, whereas structural degradation of the NCM cathode becomes the major failure source at SOCs. Overall, chemical and electrochemical interfacial degradation are strongly coupled and evolve synergistically during battery operation. Their interplay continuously reconstructs the cathode/electrolyte interface, disrupts ion transport, and constitutes a major barrier to long‐term cycling and high‐voltage operation in SSBs.

#### 2.2.2 Interfacial Side Reactions and Li Dendrite Growth in Anodes

The severe chemical potential mismatch between the SSE and the anode directly drives the formation of an interphase through redox decomposition, chemical reactions, and electrochemical decomposition (Figure 4a) [119]. Redox decomposition involves the insertion or extraction of electrons and mobile cations (such as Li^+^ and Na^+^) within the SSE upon contact with the electrode. Such reactions can be partially reversible, as demonstrated by the reversible oxidation and reduction in Li_10_GeP_2_S_12_ SSBs [120]. In contrast, chemical reactions involve the formation of new phases driven by the combined compositional space of the SSE and the anode. These redox and chemical processes often occur simultaneously or sequentially and dictate the overall interfacial stability. Electrochemical decomposition is strictly governed by the intrinsic electrochemical stability window of the SSEs. Figure 4b summarizes the electrochemical stability windows of common electrolytes and their interfacial compositions in equilibrium with Li metal [121, 122]. SSEs containing easily reducible metal cations, such as most halides, Ti in NASICONs and perovskites, or Ge in LISICONs and Li_10_GeP_2_S_12_ are particularly vulnerable to reduction at low potentials [123, 124, 125]. When their resulting decomposition layer forms an alloy and acts as a mixed ionic and electronic conductor, it cannot passivate the SSE surface. Instead, it sustains continuous parasitic reactions and leads to an ever‐growing interphase thickness, unmanageable internal resistance, and eventual short circuits. However, interfacial decomposition does not inherently preclude battery functionality provided the resulting interphase is electronically insulating. For instance, the reduction of SSEs like LiPON, Li_3_PS_4_, and Li_6_PS_5_Cl against Li metal yields electronically insulating binary Li compounds, including Li_2_O, Li_2_S, Li_3_P, Li_3_N, and LiCl [126]. These decomposition products form a passivating SSE interphase that is thermodynamically stable against the high Li chemical potential of the anode, which effectively extends the apparent cathodic limit of the SSE (Figure 4c) [106]. By eliminating the thermodynamic driving force for further reduction, this passivation mechanism yields an interphase of finite thickness, thereby arresting continuous degradation while contributing a stable and constant impedance to the full cell.

**FIGURE 4:**
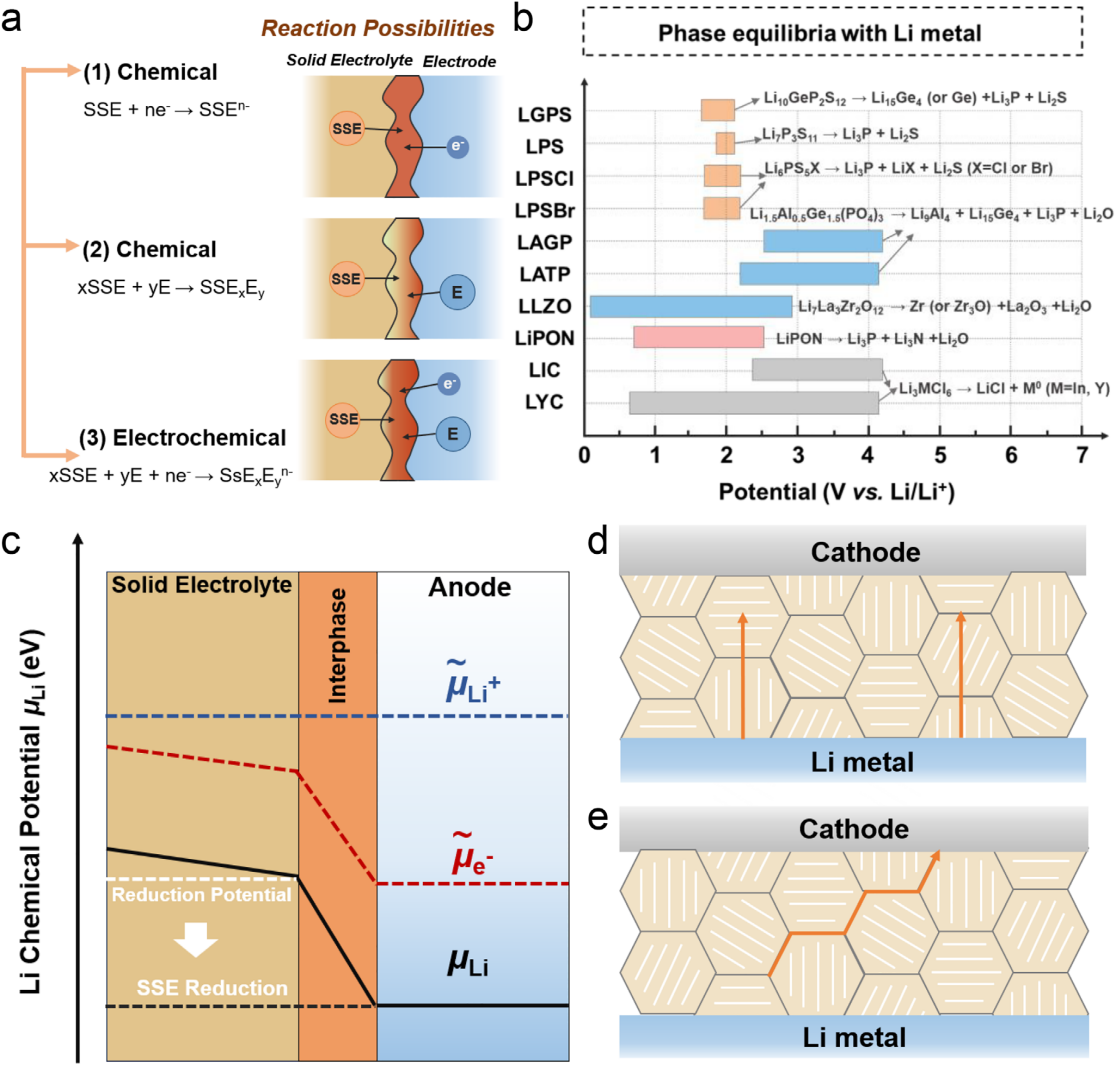
(a) Illustration of the parasitic redox, chemical, and electrochemical reactions at the SSE/Li interface. Adapted with permission [119]. Copyright 2019, Springer Nature. (b) Schematic diagram of the change of Li chemical potentials µ_Li_, the electrochemical potential μ̃_Li+_, and μ̃_e−_ across the interface between the anode and the SSE. (c) The electrochemical stability windows of common electrolytes and their interfacial compositions in equilibrium with Li metal. Adapted with permission [106]. Copyright 2010, American Chemical Society. (d) *Trans*‐granular‐type Li dendrite growth mechanism. (e) Intergranular‐type Li dendrite growth mechanism.

The uneven distribution of interphase products also promotes Li dendrite growth. Accumulation of reaction products and local charge transfer hindrance elevate the overpotential at interface defects or weak points, driving preferential Li nucleation and deposition. Dendrite growth exposes additional interfacial regions, accelerating SSE decomposition and further destabilizing the interface [127]. This coupling effect leads to a low critical current density of typically less than 1.5 mA cm^−2^ in sulfide SSEs, facilitating dendrite formation and short circuits. For polymer SSEs such as PEO with LiTFSI, nonuniform interphase structures cause uneven Li^+^ flux and result in uneven deposition [128]. Additionally, their low Li^+^ transference numbers result in SCL formation at the interface, enhancing local electric fields and promoting dendrite nucleation. This type of dendrite generation predominantly originates at the Li/SSE boundary and directly penetrates the SSE, triggering short‐circuit failures (Figure 4d) [18]. Furthermore, SSEs with relatively high electronic conductivity, such as Li_7_La_3_Zr_2_O_12_ or Li_3_PS_4_ are prone to generating Li dendrites internally [129]. They allow Li^+^ reduction along internal pathways or grain boundaries, forming interconnected internal dendrites that propagate through the electrolyte bulk (Figure 4e). In contrast, SSEs with low electronic conductivity, such as LiPON, effectively suppress internal dendrite formation and enhance cycling safety. Overall, Li dendrite formation in SSBs is a consequence of multifield‐coupled failure driven by the interplay among local physical inhomogeneity, interfacial chemical reactions, and electrochemical processes.

### 2.3 Understanding of (Electro)Chemical‐Mechanical Failure Coupling

Interfacial failure in SSBs is not governed by a single mechanical, chemical, or electrochemical factor, but results from the continuous coupling and synergistic evolution of all three [130]. This degradation process is essentially a cooperative instability involving structural damage at the interface, compositional evolution, and imbalance in local reaction kinetics. Repeated volume expansion and contraction of electrode active materials during cycling continuously build up stress at the interface, leading to mechanical damage such as particle cracking and void formation. These structural defects not only directly disrupt continuous ion‐transport pathways but also expose a large amount of highly reactive fresh surface, creating favorable conditions for subsequent interfacial side reactions [131]. In other words, mechanical damage strongly accelerates chemical degradation. To better illustrate this coupled degradation process, Figure 5 summarizes the electro‐chemo‐mechanical feedback loop at SSB interfaces. This coupling is especially evident in Si‐based anodes. Because Si undergoes dramatic volume changes of approximately 300% during (de)lithiation, the interfacial reaction layer can easily fracture and become mismatched under repeated stretching and contraction, exposing new Si/SSE contact areas and triggering local re‐reactions [99]. Similarly, the initiation of Li dendrite growth in SSBs is widely attributed to inhomogeneous mechanical contact. Uneven Li stripping and plating at localized point contacts inevitably lead to void formation at the Li/SSE interface, accompanied by a drastic reduction in contact area and severe local stress redistribution. As stress‐induced microcracks emerge, they expose fresh interfaces, inducing the continuous formation of new solid electrolyte interface (SEI) between the highly reactive Li and the electrolyte [132]. During prolonged cycling, the progressive accumulation and thickening of this SEI severely disrupt interfacial Li^+^ transport kinetics. This kinetically sluggish environment further aggravates localized Li deposition and dendrite penetration, ultimately culminating in a battery short circuit. Therefore, mechanical damage accelerates chemical decay through fresh surface exposure and local kinetic deterioration.

**FIGURE 5:**
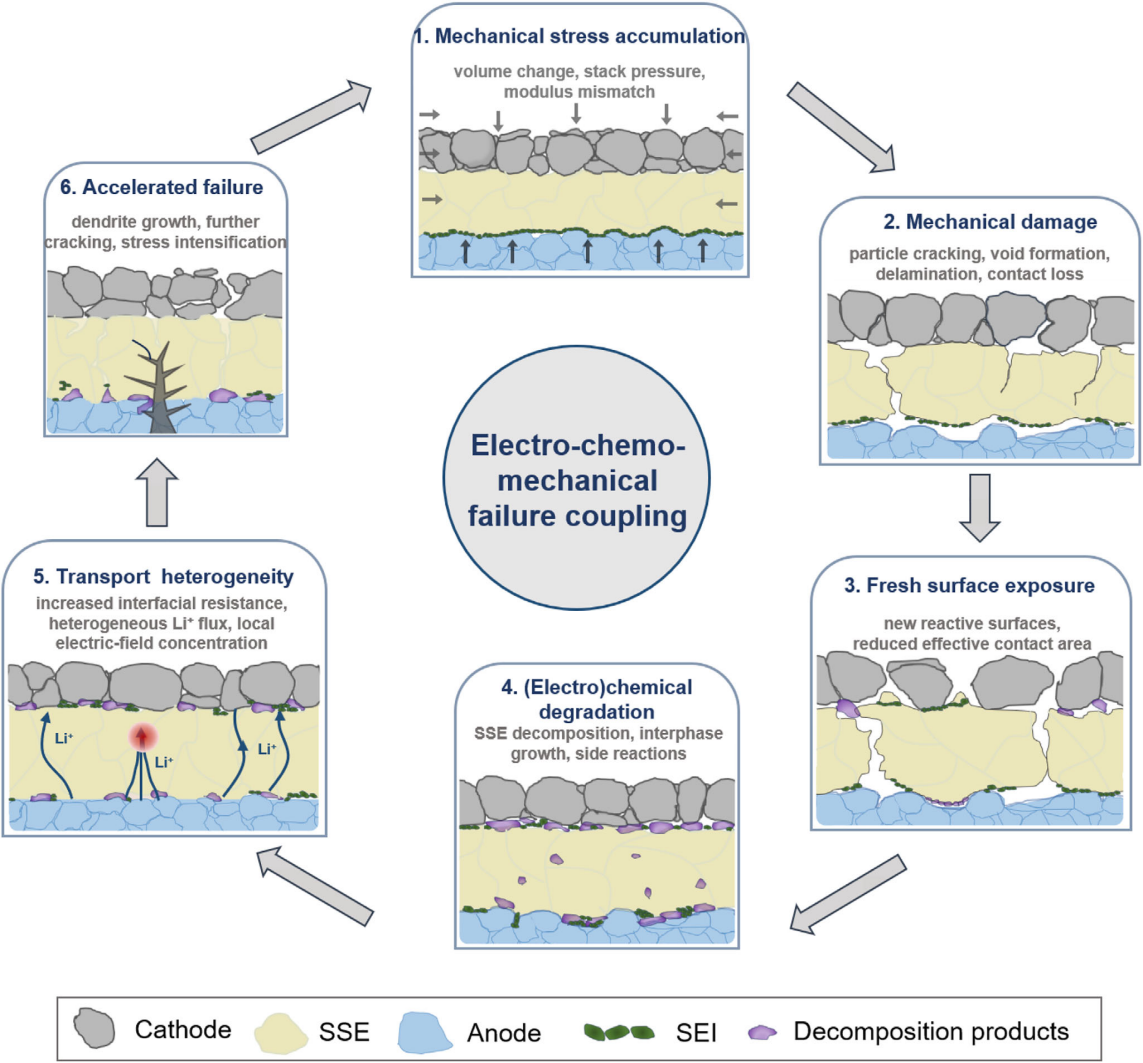
Conceptual schematic of the electro‐chemo‐mechanical feedback loop at solid‐state battery interfaces.

In turn, interfacial chemical evolution can further weaken the interfacial mechanical integrity, forming a continuously self‐amplifying failure loop. As chemical or electrochemical decomposition proceeds, the interface continuously generates Li_2_S, P_2_S_x_, Li_3_P, and other reduced or oxidized byproducts [133]. These reaction products show poor ionic transport capability, and their structure and mechanical properties differ from those of the original electrode or SSE. As a result, they introduce new modulus mismatch and local stress concentration at the interface. Operando Raman and interfacial chemistry studies have shown that sulfide SSEs can form complex decomposition layers when in contact with Li metal or cathodes, and these products continuously alter local bonding states and microstructural evolution [134]. More recent studies suggest that chemically driven reaction kinetics can directly determine the subsequent microstructural evolution, indicating that chemical degradation itself reshapes the pathway of mechanical deterioration [135]. Ultimately, pervasive chemical and electrochemical side reactions severely exacerbate the physical degradation of the interface, accelerating complete contact failure.

Although mechanical, chemical, and mechanochemical degradation mechanisms are highly coupled, the dominant failure mode often varies depending on the specific electrochemical conditions and material configurations. For instance, recent studies have explicitly demonstrated this dynamic transition. In the case of NCM811, Zheng et al. found that capacity decay in the bare cathode is primarily driven by electrochemical degradation [136]. However, once a LiNbO_3_ coating effectively suppresses interfacial side reactions, mechanical degradation, including particle cracking and contact loss, emerges as the more prominent limiting factor [137]. This finding indicates that interfacial coatings do not universally suppress all failure mechanisms, but can instead shift the dominant degradation pathway. Furthermore, when the nickel content is increased to 85%, the decisive factor limiting battery life fundamentally transitions to mechanically induced failure, including severe particle isolation and delamination from the electrolyte [138]. Therefore, while the overall failure of these materials is undeniably the result of complex mechanical‐chemical‐electrochemical coupling, systematically identifying the dominant degradation mechanism at specific stages is crucial for developing targeted interventions and extending the lifespan of SSBs.

## 3 Interfacial Self‐Healing Strategies for SSBs

To address the coupled (electro)‐chemical‐mechanical failure, researchers have traditionally employed strategies such as elemental doping or surface coating to enhance the electro‐chemo‐mechanical stability of materials and their interfaces [139]. Despite their proven efficacy in delaying initial degradation, these mitigation strategies are rendered entirely defenseless once interfacial failure commences. Once an internal defect, such as a micro‐crack, originates, it tends to propagate uncontrollably, leading to rapid performance decay. Consequently, engineering dynamic, self‐healing behaviors within the battery architecture emerges as a vital imperative [140]. By autonomously addressing and resolving microscopic damage at its inception, self‐healing mechanisms enable the interface to sustain its original protective state and structural integrity. In this section, we systematically summarize emerging interfacial self‐healing technologies, categorizing them into physical flow filling, dynamic bonding networks, and field‐assisted healing based on their underlying repair principles. These dynamic approaches aim to mitigate damage in real time, thereby significantly extending the cycle life of SSBs.

### 3.1 Physical Flow and Space‐Filling

Physical flow and space‐filling interfacial self‐healing behavior exploits the stress‐induced flow, rearrangement, and interfacial spreading of materials to actively fill and bridge physical gaps generated during electrochemical cycling [141]. This strategy is particularly important for SSBs, where it maximizes effective interfacial contact in a manner akin to the wetting behavior of traditional liquid electrolytes. Current efforts have mainly focused on two representative materials, including viscoelastic polymers and low‐melting‐point alloys, offering a promising route to mitigate interfacial contact degradation and mechanical instability arising from volume variation and stress accumulation during cycling.

#### 3.1.1 Creep and Filling of Viscoelastic Polymers

Viscoelastic polymers are widely used in various battery components as binders, electrolyte matrices, and interfacial coatings due to their creep and stress‐relaxation behavior [142, 143]. These polymers are typically designed with a glass transition temperature (Tg) below room temperature, allowing dynamic motion of long polymer chains to bridge physical gaps caused by volume fluctuation. When electrode active materials undergo volume contraction upon de(lithiation), micropores are formed, leading to interfacial contact loss and even local delamination. Under such conditions, polymer segments in the rubbery or viscous state can spontaneously migrate toward regions of stress release under internal or external stress [144]. This process is essentially driven by the rearrangement and re‐spreading of polymer chains, thereby enabling physical repair of interfacial defects.

Fluorinated copolymers, such as poly(vinylidene fluoride‐co‐hexafluoropropylene) (PVDF‐HFP) serve as highly representative viscoelastic polymers [145]. Due to the incorporation of amorphous HFP units, these polymer matrices exhibit significantly reduced crystallinity and enhanced chain mobility. Under the continuous mechanical stress generated by the volume fluctuations of active materials, the PVDF‐HFP matrix can undergo physical deformation and viscous flow. Lian et al. developed a 3D crosslinked single‐ion‐conducting polymer electrolyte, LiPHB, from the boron‐centered Li salt LiT_4_PAB and PVDF‐HFP [146]. Benefiting from rich conformational transitions, LiPHB undergoes adaptive network deformation, achieving a relatively high modulus of 1.4 GPa and a high elongation of 447%. Meanwhile, the construction of 3D Li^+^ transport pathways enable favorable interfacial stability and long‐term cycling performance. However, the segmental dynamics at room temperature are limited in pure polymer systems. Plasticizing the matrix with highly polar additives like succinonitrile (SN) or integrating inorganic SSEs into these dynamic polymer networks not only accelerates the physical flow for rapid interfacial healing but also further enhances the overall mechanical strength of the composite [147]. A composite electrolyte with self‐healing capability, flowability, and solid‐state characteristics was recently reported [148]. The initial polymer phase consists of a polyacrylate backbone, an ionic liquid plasticizer, and LiFSI salt. After further incorporation of the Li_6_PS_5_Cl SSE, the resulting composite can dynamically switch between liquid‐like flow response and solid‐like load‐bearing behavior depending on the applied strain rate. In this architecture, the dynamic polymer network enables crack closure, while the migration of movable ceramic crystals further fills the voids, achieving a high self‐healing rate of 5.6 µm h^−1^ (Figure 6a). This strong flowability accommodates the volume change of Li metal during stripping, while maintaining a high tensile strength of 0.28 MPa, effectively regulating the deposition morphology of mossy Li. Similarly, other highly flexible matrices, such as poly(ethylene oxide) (PEO) and polydimethylsiloxane (PDMS), leverage their exceptional chain mobility at operational temperatures to maintain intimate conformal contact, thereby continuously repairing structural voids and homogenizing the interfacial ion flux [149]. Overall, these viscoelastic polymers are commonly used as electrolyte components in solid‐state batteries. Their excellent deformability makes them particularly suitable for self‐healing within polymer‐based electrolytes and for repairing electrode/electrolyte interfacial contact. In general, viscoelastic polymer matrices show relatively good compatibility with anode interfaces, where their strong reducing resistance and flexibility help suppress Li dendrite growth. Their applicability at cathode interfaces, however, strongly depends on their electrochemical stability window. For example, PVDF‐HFP‐based matrices can usually tolerate relatively high voltages, whereas PEO‐based systems are more difficult to pair with high‐voltage cathodes because of their limited oxidative stability.

**FIGURE 6:**
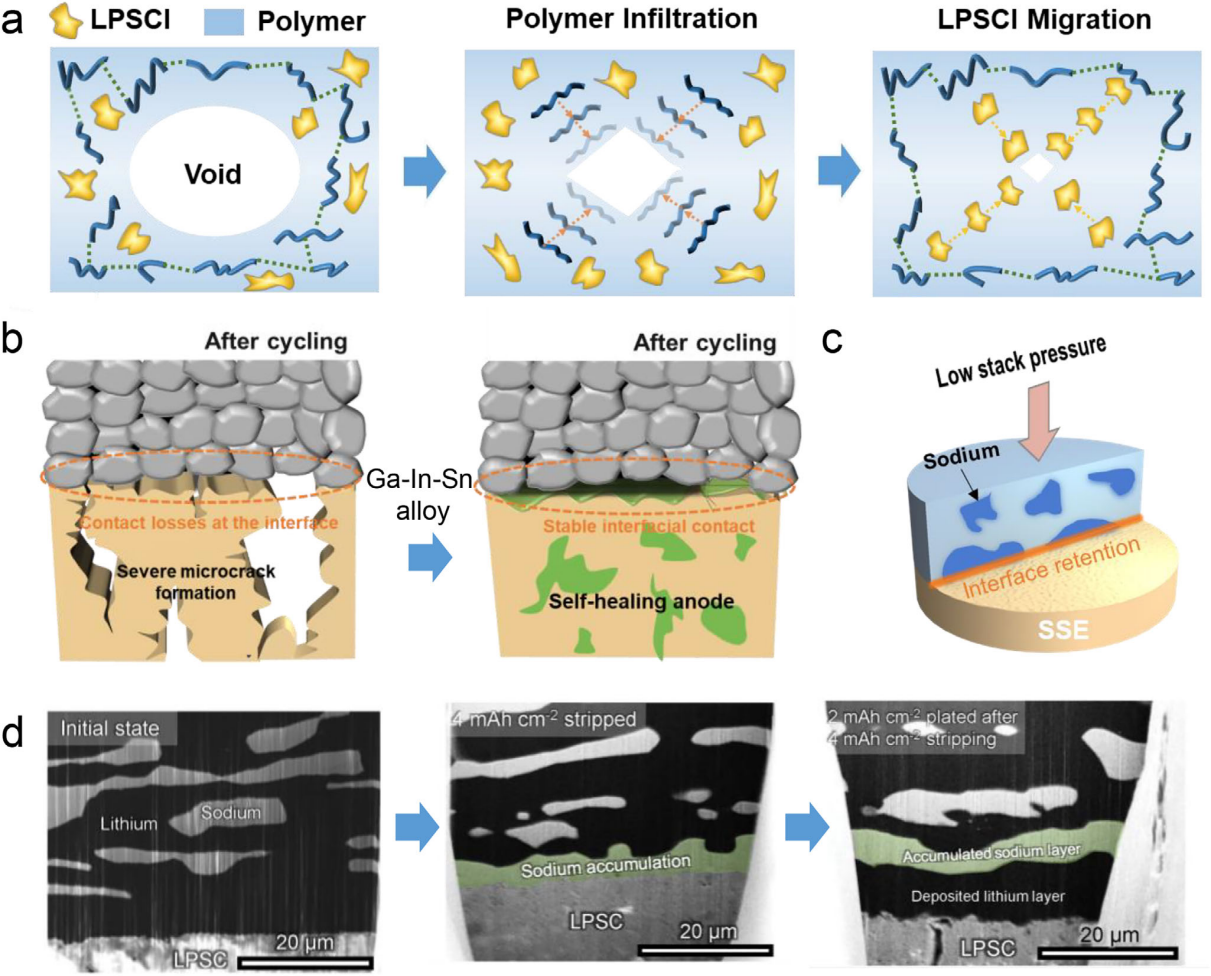
(a) Schematic diagram illustrating the mechanism of the synergistic filling of voids by organic polymers and inorganic SSEs. Adapted with permission [148]. Copyright 2010, American Chemical Society. (b) Schematic diagram showing the use of Ga–In–Sn alloy to fill the cracks generated in the Si anode. Adapted with permission [155]. Copyright 2023, Elsevier. (c) Schematic diagram of a dynamically adjustable soft buffer Li‐Na layer that fills voids. Adapted with permission [156]. Copyright 2025, American Association for the Advancement of Science. (d) Cryo‐FIB‐SEM images of 10% Na electrodes at various stages of cycling: the initial state, after 4 mA·hour cm^−2^ was stripped, and after 2 mA·hour cm^−2^ was plated. Reproduced with permission [156]. Copyright 2025, American Association for the Advancement of Science.

Beyond bulk composite electrolytes, viscoelastic polymers can also enable localized self‐healing in the form of interfacial adhesive layers. For example, a dual interfacial engineering strategy combines nanoengineered Li_6+x_PS_5+x_Cl_1−x_ SSE with an adaptive polyethylene‐co‐vinyl acetate/Li difluoro(oxalato)borate composite adhesive coating on layered oxide cathodes [32]. This design enables stable interfacial integrity and continuous ionic transport networks under low stack pressure. Through physical gap filling and stress buffering, viscoelastic polymers suppress the sharp increase in interfacial resistance caused by contact loss and avoid local stress concentration via stress redistribution [150]. When used as interfacial modifiers in cathodes or anodes, their practical applicability should be carefully evaluated based on their intrinsic redox stability, as insufficient oxidative or reductive tolerance may induce additional interfacial side reactions. With appropriate chemical stability, such polymer‐based interfacial modifiers are expected to reduce the dependence of SSBs on high external stack pressure, lowering the required pressure from tens of MPa to the single‐digit MPa range. This feature is of great importance for the practical development of low‐pressure SSBs.

#### 3.1.2 Adaptation of Low‐Melting‐Point Alloys and Metals

If viscoelastic polymers mainly rely on chain‐segment migration to heal interfacial defects, low‐melting‐point metals and alloys can further enable faster and more direct physical self‐healing due to their higher flowability, better wettability, and stronger plastic deformability [151]. Low‐melting‐point In‐based and Ga‐based alloys, as well as soft metals with low modulus, have emerged as another important class of interlayer materials for maintaining the physical integrity of interfaces in SSBs [152]. These materials usually exist in liquid, semisolid, or slushy states near room temperature, combining high surface energy, excellent wettability, and nearly unlimited deformability. They spontaneously flow during electrochemical cycling and continuously fill newly formed physical defects in real time. This feature is particularly useful for mitigating void formation, delamination, and contact failure on the anode side during stripping and plating.

In Li‐based SSBs, the flowability of low‐melting‐point alloys can directly compensate for interfacial shrinkage induced by Li stripping. Cui's group embedded a ductile (InSn_4_)_0.37_·(InBi)_0.63_ alloy into a high‐area inert arched Ti mesh [153]. Owing to its low melting point, low modulus, and high rheological activity, the alloy can continuously fill interfacial defects and maintain bicontinuous ionic/electronic pathways under pressures below 10 MPa. Meanwhile, the arched Ti mesh effectively disperses local stress and suppresses the propagation of creep‐induced instability. In this way, the volume expansion of high‐capacity alloy anodes, which is usually regarded as a failure source is converted into a driving force for interfacial self‐healing. GaInSn‐type Ga‐based liquid metals exhibit similar self‐healing behavior due to their liquid nature at room temperature and good wettability. Luo et al. prepared a Ga‐In‐Sn‐Li low‐melting‐point alloy interlayer in situ at the Li/LATP interface [154]. By improving the contact between the Li anode and SSE, this interlayer markedly alleviated interfacial mismatch and impedance growth during cycling. Beyond stabilizing Li metal, the exceptional fluidity of these liquid alloys is particularly advantageous for addressing the severe volumetric fluctuations of high‐capacity Si anodes [155]. Through the Ga‐In‐Sn alloy reversible solid‐liquid phase transition in the Si anode, these alloys can continuously fill cracks and preserve ionic/electronic conductive networks, endowing high‐capacity Si electrodes with clear self‐healing characteristics (Figure 6b).

In addition to low‐melting‐point liquid metals, soft metals and their alloys with low yield stress can also realize dynamic interfacial repair through plastic flow. A representative example is a Li‐Na composite metal foil formed by dispersing micron‐sized Na within a continuous Li matrix [156]. As shown in Figure 6c, during electrochemical stripping, the dispersed Na‐rich domains spontaneously migrate toward the SSE interface. Because of their extremely low yield strength (0.16‐0.24 MPa), these domains undergo pronounced plastic deformation and form a dynamically adjustable soft buffer layer at the interface. Cryo‐FIB‐SEM revealed the dynamic redistribution of the Na‐rich phase in the Li‐Na composite anode. Initially, Na domains were uniformly dispersed within the Li matrix and did not contact the SSE. After Li stripping, Na migrated toward the Li/SSE interface and formed a several‐micrometer‐thick Na‐rich layer, which served as a soft buffer to compensate for Li depletion and maintain interfacial contact. Upon subsequent Li deposition, this Na‐rich layer shifted away from the interface, demonstrating reversible interfacial adaptation (Figure 6d). This layer continuously preserves electrical contact and suppresses the nucleation and growth of voids, enabling stable cycling under an ultralow stack pressure below 1 MPa. The HC‐Sn phase with relatively high Li^+^ diffusivity is subsequently introduced into the Li‐Na anode, and the Li/SSE evolves in situ into a Li_6_PS_5_Cl/[HC‐Li_x_Sn]Na/Na/LiNa multilayer structure after Li deposition [157]. The new structure not only guides uniform Li deposition at the target interface and suppresses local current concentration, but also the plastic flow of the soft Na phase during stripping helps relieve void formation. Both interfacial morphology regulation and contact retention are achieved simultaneously.

In Na‐based systems, liquid Na‐K alloys provide an even more representative model of flow‐enabled self‐healing. Cheng et al. constructed a carbon‐fiber‐supported liquid Na‐K alloy anode [158]. Benefiting from its excellent flowability and interfacial wettability, the alloy dynamically compensates for contact loss and gap formation at the solid‐solid interface during Na plating and stripping, reducing interfacial resistance and improving cycling stability. However, when the highly reactive Na‐K liquid alloy is in direct contact with the NASICON SSEs, the penetration and conversion reaction of K^+^ triggers a severe collapse of the SSE crystal structure. To overcome this, Guo's group introduced a flexible, charge‐selective polymer thin layer (PEO‐NaClO_4_) at the interface [159]. Governed by solubility equilibrium and electrochemical kinetic control, this coating successfully blocks the penetration of harmful K^+^ while maintaining stable Na^+^ charge transport. Therefore, coupling the physical flow‐filling of liquid metals with a dynamic chemical and kinetic protective layer is highly advantageous for achieving long‐term self‐healing and interfacial stability in SSBs. Overall, interfacial self‐healing strategies based on low‐melting‐point alloys, liquid metals, and soft‐metal matrices are mainly used to improve anode/electrolyte interfaces or serve as functional components on the anode side. This preference is closely related to their intrinsic electrochemical properties. Under high‐voltage cathode conditions, these metallic or liquid‐metal components are prone to oxidation and may introduce undesirable side reactions. In contrast, at low‐potential anode interfaces, they can participate in reversible alloying reactions with Li or Na, regulate Li^+^/Na^+^ deposition behavior, and accommodate large volume fluctuations during repeated plating/stripping. Therefore, they move beyond the traditional concept of passively resisting interfacial failure through high mechanical strength alone. By taking advantage of low yield stress, high flowability, and reversible phase transitions, these materials can actively compensate for volume change and continuously reconstruct the interface during cycling.

### 3.2 Dynamic Chemical Bonding and Supramolecular Interaction

While physical flow‐filling provides a macroscopic strategy to accommodate interfacial gaps, dynamic chemical bonding and molecular interaction introduce an elegant paradigm of self‐healing at the molecular level [160]. During the severe volume expansion and contraction of electrode materials, these dynamic bonds function as sacrificial linkages. They reversibly dissociate to dissipate localized mechanical stress, preventing catastrophic matrix failure, and subsequently re‐associate to autonomously stitch microscopic cracks. By embedding these dynamic networks into polymer SSEs or interfacial binders, the electrode/electrolyte interface gains a unique balance of mechanical toughness and spontaneous repairability [161]. This section systematically reviews the application of various dynamic chemistries in SSBs, elucidating how molecular‐level reversibility translates into macroscopic interfacial stability.

#### 3.2.1 Reversible Covalent Bonding

Harnessing dynamic covalent bonds for self‐healing offers a robust pathway to overcome the mechanical mismatches and ionic transport instabilities inherent in polymer‐incorporated SSBs during prolonged cycling. These bonds can undergo reversible breaking and reformation under stimuli such as heat, stress, or changes in the local chemical environment, offering the dual functions of mechanical support and damage repair [160]. The self‐healing process is generally characterized by three successive stages: stress response, network reconstruction, and property recovery [162]. First, initially, integrating polymers equipped with dynamic covalent networks into battery components ensures seamless ion transport across all interfaces, maintaining normal battery operation (Figure 7a). During subsequent electrochemical cycling (Figure 7b), when substantial stress is generated by the volume expansion and contraction of electrode active materials, dynamic covalent bonds embedded within the polymer network act as sacrificial linkages. They preferentially break to dissipate mechanical energy through reversible bond cleavage, preventing stress concentration from developing into penetrating cracks. The composite then enters a stage of chain rearrangement and dynamic exchange, where active functional groups near the damaged interface rebuild chemical connections through exchange reactions. This process drives segmental motion and network topology rearrangement, enabling molecular‐level stitching of the interface. Finally, as the crosslinked network reconstructs, both the mechanical continuity of the interfacial layer and the ionic transport pathways are restored (Figure 7c). This property recovery effectively suppresses the capacity decay typically caused by crack propagation and localized delamination. This dynamic healing mechanism has been widely validated across various dynamic covalent systems, including imine bonds, boronate ester bonds, and disulfide bonds with their specific restorative mechanisms explicitly illustrated in Figure 7d [163].

**FIGURE 7:**
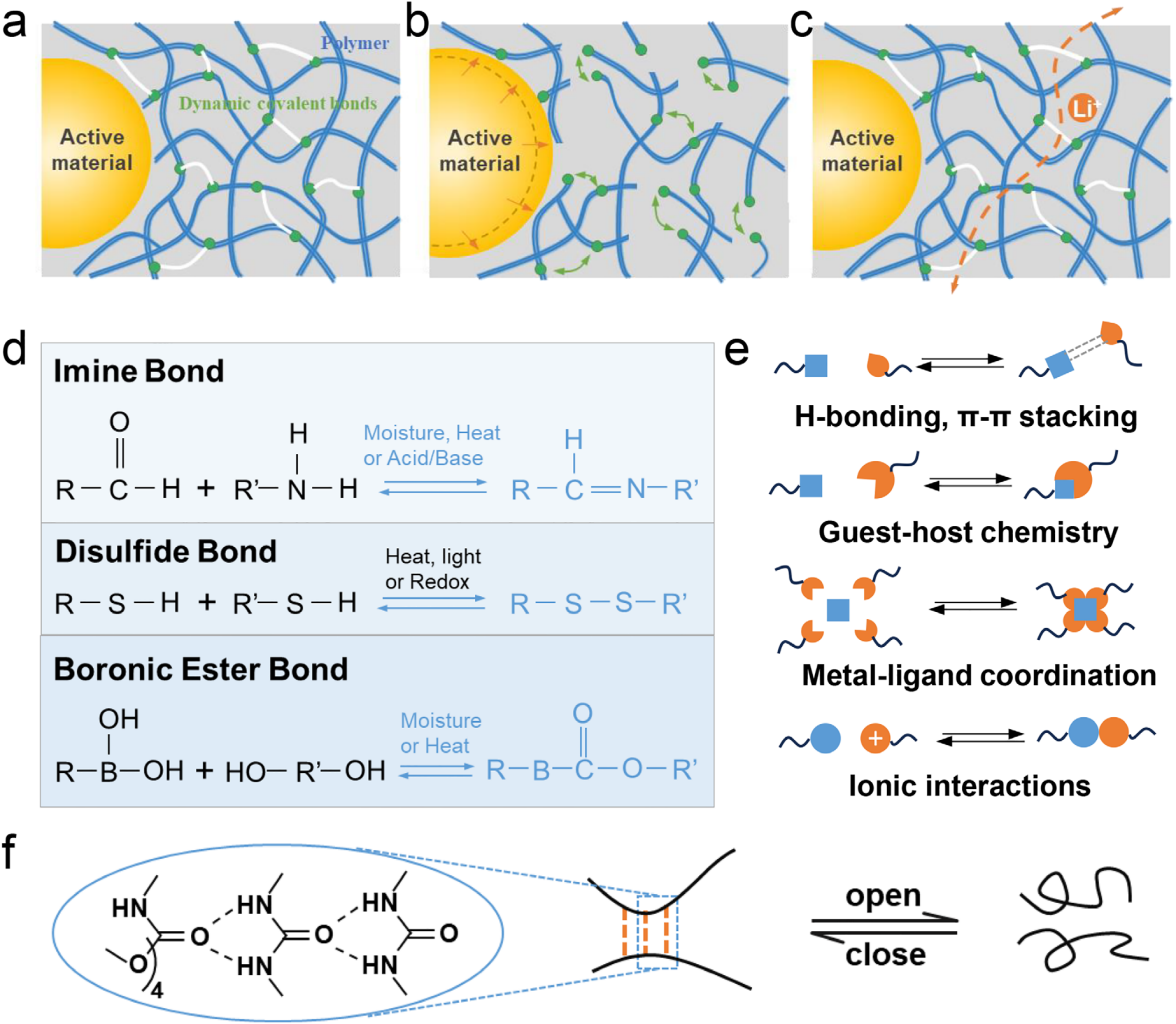
(a–c) The mechanism of polymers with dynamic covalent bonds for repairing interface damage. (d) The healing mechanism of typical dynamic covalent bonds. (e) Schematic diagram of supramolecular chemistry. (f) The self‐repair mechanism of the hydrogen bond formed by the amino groups.

Among dynamic covalent polymers, imine bonds represent one of the most prominent building blocks for SSE matrices. Cao et al. reported a Schiff base/imine bond‐based self‐healing polymer SSE, showing that the reversible formation and cleavage of imine bonds endow the material with pronounced self‐healing ability and interfacial stability while maintaining relatively high mechanical strength [164]. Similarly, a dynamic imine vitrimer SSE developed by Yang et al. exhibited distinct stress relaxation and room temperature healing characteristics [165]. An initial decrease in interfacial resistance was observed during cycling, suggesting that imine exchange reactions not only facilitate topological network rearrangement but also enhance electrode/electrolyte compatibility and continuous ion transport. At the electrolyte level, the role of dynamic covalent bonds extends far beyond simple macroscopic crack repair. Instead, through segmental motion, bond exchange, and reconnection, they actively reintegrate locally disconnected interfaces into a continuous ion‐conducting network. Similar dynamic self‐healing behavior has been widely reported for boronate ester‐based polymer SSEs, where dynamic transesterification continuously adjusts the network topology, enabling both effective self‐healing and enhanced ductility [166]. To synergize the dynamic healing ability of polymers with the high ionic conductivity and mechanical rigidity of inorganic SSEs, researchers have further integrated dynamic covalent networks into organic/inorganic composite electrolytes. For example, a flame‐retardant and self‐healing polyurethane‐thiourea polymer was chemically grafted onto Li_7_P_3_S_11_ via nucleophilic addition to fabricate a composite electrolyte (SHPUSB‐40%LPS) [31]. This composite demonstrated excellent ionic conductivity, high elasticity, and strong compatibility with Si anodes. In this system, dynamic covalent disulfide bonds and hydrogen bonds enable the electrolyte to self‐assemble into a re‐constructable network at the interface. Concurrently, FSI^−^ anions are adsorbed on the LPS surface through electrostatic interaction with sulfur vacancies, further enhancing Li^+^ transport. These multiple dynamic interactions provide the battery with continuous interfacial healing capability, effectively mitigating structural damage induced by repeated volumetric expansion and contraction of Si.

Beyond their role as SSE matrices, polymers featuring dynamic covalent bonds also serve as highly effective binders or interfacial adhesive layers to stabilize interparticle contact. A self‐healing supramolecular adhesive additive integrated with dynamic covalent disulfide bonds has been reported to deliver outstanding elasticity, with a deformation exceeding 2300%, and fast room‐temperature self‐recovery [167]. During cell operation, in situ fiber‐optic sensing confirmed that this adaptive network effectively buffers the lattice stress of layered oxide particles and autonomously repairs interfacial damage, thereby continuously maintaining interparticle contact and the integrity of Li‐ion transport pathways. Crucially, dynamic covalent networks do not simply force a trade‐off between rigidity and healability. Through rational molecular design, they achieve an optimal balance between high‐modulus mechanical support and autonomous damage repair. Dynamic covalent bond‐based polymer SSEs are particularly suitable for healing internal defects within flexible polymer electrolyte matrices and repairing anode/polymer electrolyte interfaces. They are especially effective in battery systems involving large volume changes, such as Li metal, Na metal, Si‐based anodes, and Li‐S batteries, where dynamic bond exchange can help dissipate stress and maintain interfacial contact. However, their application at high‐voltage cathode interfaces requires careful consideration of oxidative stability. Overall, compared to healing mechanisms that primarily rely on physical polymer flow or viscoelastic migration, networks formed by dynamic covalent bonds typically exhibit greater structural stability and modulus. As a result, they not only enable robust self‐healing but also more effectively suppress creep, excessive deformation, and fatigue failure during extended cycling.

#### 3.2.2 Supramolecular Interaction

Supramolecular interactions, including hydrogen bonding, guest‐host chemistry, metal‐ligand coordination, ionic interactions, and van der Waals forces can construct highly adaptive dynamic networks at SSB interfaces owing to their low activation energy and rapid kinetic responses (Figure 7e) [168]. Compared to dynamic covalent bonds, supramolecular interactions undergo rapid dissociation and reformation under significantly milder conditions, often relying on only mild thermal or chemical stimuli to trigger exchange reactions. This exceptional reversibility makes them particularly well‐suited for mitigating the continuous accumulation of microscale damage during prolonged SSB cycling.

Among various supramolecular interactions, hydrogen bonding is a representative driving force for constructing self‐healing solid interfaces, benefiting from its strong directionality, rapid reversibility, and dynamic activity [169]. When severe electrode volume changes or interfacial stress accumulation induce microcracks and local delamination, polar functional groups (such as amino groups) exposed on the fractured surfaces rapidly re‐establish hydrogen bonds through strong molecular recognition and affinity (Figure 7f) [170]. This spontaneous process triggers reversible recombination, enabling the real‐time repair of physical defects, suppressing further crack propagation, and maintaining continuous ionic transport pathways alongside intimate electrode/electrolyte contact. A typical example is the polymerized ionic liquid‐based self‐healing polymer SSE reported by Lin et al. [171]. This electrolyte employs a poly (methyl methacrylate) (PMMA) matrix, into which imidazolium‐based ionic liquid moieties (EMIM^+^) are grafted via free‐radical polymerization, with TFSI^−^ as the counter anions. Such a design integrates the high ionic conductivity and electrochemical stability of ionic liquids with the robust mechanical properties of polymer backbones. Strong intermolecular hydrogen‐bonding interactions between the C‐H groups of the imidazolium cations and the carbonyl oxygen in PMMA chains endow the electrolyte with intrinsic self‐healing capability. Driven by this dynamic network, this system spontaneously reconstructs dendrite‐induced defects and fatigue cracks at the Li/SSE interface at room temperature, thus improving interfacial stability and cycling life.

While a standalone hydrogen‐bonding network exhibits rapid responsiveness and commendable reversible healing, it inherently suffers from limitations in mechanical strength, long‐term structural stability, and load‐bearing capacity under rigorous operating conditions. To resolve this, researchers have increasingly combined hydrogen bonding with dynamic covalent bonds to engineer hierarchical, synergistic self‐healing networks. Such networks are more suitable for repairing internal defects within flexible polymer SSE and anode/polymer electrolyte interfaces. For instance, Pei and co‐workers developed a polyether‐urea‐based polymer electrolyte featuring a cooperative network of dynamic disulfide and hydrogen bonds [160]. This work explicitly demonstrates that hydrogen bonds not only endow the polymer network with conformal adaptability and rapid stress‐dissipation capabilities but also act synergistically with dynamic covalent units to fortify the overall mechanical integrity of the interfacial layer. Consequently, stable solid‐solid contact is continuously preserved, and progressive defect accumulation is effectively suppressed. In these cooperative architectures, hydrogen bonds primarily facilitate instantaneous stress response and localized network reconstruction, whereas dynamic covalent bonds deliver robust structural support and enduring healing efficacy, achieving an optimal equilibrium between rapid interfacial recovery and long‐term cyclic stability.

### 3.3 External‐Field Coupled Smart Responsive Mechanisms

Beyond self‐healing strategies that rely on the intrinsic dynamic properties of materials, external physical fields offer another important route to improve interfacial stability in SSBs. Compared with spontaneous healing by the material itself, external field‐assisted strategies actively regulate interfacial evolution through external energy input [172]. This approach shows unique advantages in mitigating contact loss, suppressing crack propagation, and maintaining continuous ion‐transport pathways. In this section, the effects of heat and mechanical force on interfacial self‐healing are mainly discussed.

#### 3.3.1 Thermally Activated Self‐Healing

Thermal field‐assisted phase‐transition strategies offer a highly targeted, on‐demand mechanism for actively reconstructing degraded interfaces in SSBs by exploiting temperature‐responsive material behavior. This approach relies on incorporating low‐melting‐point crystalline components or thermally responsive polymers into the interfacial architecture [173]. In principle, these components can be designed for different interfaces in SSBs, including cathode/SSE, anode/SSE, and internal composite‐electrode interfaces. However, a fundamental prerequisite is that these functional components must not compromise intrinsic ion transport kinetics, either before or after thermal activation. When the local interfacial temperature approaches or exceeds the melting transition point, triggered either by current‐induced local hot spots or by controlled external heating, these responsive components undergo instantaneous localized melting or softening. This phase transition imparts a crucial degree of flowability and stress‐relieving capability to the interface, allowing for adaptive structural reconstruction under mechanical strain. In this way, mechanical defects are effectively targeted and repaired, interfacial gaps are bridged, and the formation of new defects is heavily suppressed, ultimately maintaining continuous Li^+^ transport pathways.

In SSBs, thermal healing primarily relies on external heat input to activate interfacial reconstruction. Specifically, targeted heating induces the localized melting of thermally responsive components, allowing them to infiltrate interfacial micro‐gaps. Upon cooling or the removal of the thermal field, these molten components recrystallize and solidify in situ. This process forms a dense structure within the defective regions, fully restoring ion‐transport pathways and reinforcing the mechanical integrity of the interface. A representative example of this strategy is S_9.3_I molecular crystal cathode active materials, synthesized by incorporating I_2_ into crystalline sulfur [174]. The introduction of iodine creates new electronic states within the sulfur band structure, significantly lowering its melting point from 112°C to approximately 65°C, while increasing its electronic conductivity to 5.9×10^−7^ S cm^−1^, an enhancement of about 11 orders of magnitude compared to pristine sulfur. This tailored modification not only improves interfacial contact and reaction kinetics but also enables controllable remelting under relatively mild thermal conditions (Figure 8a). Experimental results demonstrate that when battery performance begins to decay, a brief thermal treatment can effectively restore capacity (Figure 8b). Correspondingly, the interfacial impedance continuously increases from the first cycle to the 50^th^ cycle, but is reduced by reheating to the value after the first cycle. A similar restoration is observed at the 100th cycle as well. (Figure 8c). This recovery originates from the redistribution of the low‐melting‐point active material, which reconnects ion and electron transport pathways. As a result, interfacial damage accumulated during cycling can be periodically repaired on demand, ensuring stable long‐term performance.

**FIGURE 8:**
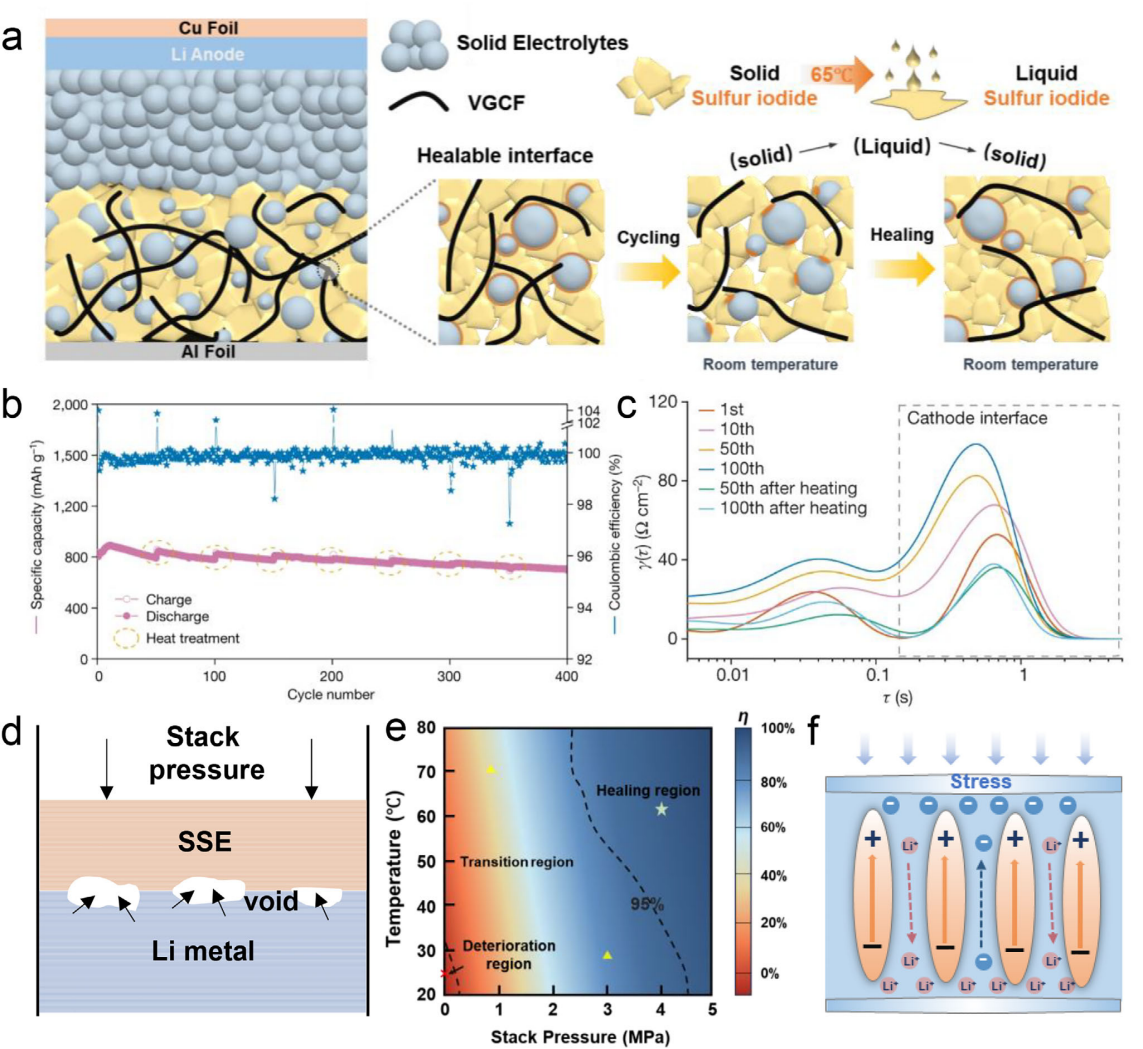
(a) Schematic diagram of thermal stimulation for repairing solid‐state lithium‐sulfur batteries with a sulfur iodide cathode. (b) Long‐term cycling performance of S_9.3_I cathode at 0.16 A g^−1^ and at 25°C. Reproduced with permission [174]. Copyright 2024, Springer Nature. (c) DRT analysis of EIS data of the Li‐S_9.3_I full cell after different cycles at 25°C. Reproduced with permission [174]. Copyright 2024, Springer Nature. (d) Schematic diagram of regulating the accumulation pressure to enable Li metal creep repair of the Li/SSE interface. Adapted with permission [176]. Copyright 2025, American Chemical Society. (e) Pressure‐temperature‐void evolution maps for the SSE/Li interface. (f) Schematic diagram of mechanical stress applied to piezoelectric materials for modulating internal battery potential. Adapted with permission [179]. Copyright 2024, American Chemical Society.

#### 3.3.2 Pressure‐Activated and Piezoelectric‐Field‐Assisted Self‐Healing

Beyond thermal stimuli, the strategic application of external stack pressure constitutes a fundamental mechanical paradigm for interfacial self‐healing in SSBs. By precisely regulating applied external pressure or strategically harnessing internally generated stresses, the interfacial architecture can actively accommodate the structural evolution and morphological changes inherent to prolonged electrochemical cycling. This stress‐induced remediation is particularly critical for metallic Li metal anodes. Extensive studies have elucidated that during the Li stripping process, voids readily nucleate at the Li/SSE interface, inevitably precipitating contact loss [175]. However, the application of an appropriate stack pressure counteracts this degradation by activating the creep deformation of the Li metal (Figure 8d). Specifically, under appropriate pressure, the soft Li metal undergoes viscoplastic flow, migrating from the bulk toward the depleted interface. This flow effectively fills the void regions, driving void shrinkage or even complete annihilation. Consequently, this mechanically forced infiltration recuperates intimate interfacial contact and drastically reduces interfacial resistance. Furthermore, sustained pressure suppresses the subsequent growth of new voids, guaranteeing the spatial continuity of Li^+^ transport. To systematically quantify this behavior, recent studies have established pressure‐temperature‐void evolution maps, meticulously defining the operating windows for optimal interfacial self‐healing (Figure 8e) [176]. Crucially, the magnitude of the applied pressure must be rigorously optimized; insufficient pressure fails to trigger adequate creep for void closure, excessive pressure can inadvertently exacerbate Li dendrite penetration, or inflict mechanical fracture upon the SSE matrix.

Beyond conventional external pressure regulation, incorporating piezoelectric materials to harness inherent internal stress has emerged as a highly effective strategy for dynamic interfacial repair [37]. It should be noted that self‐healing based on piezoelectric materials differs from conventional pressure‐induced repair. In pressure‐assisted Li creep or soft‐material deformation, external mechanical force directly drives void closure or contact recovery. In contrast, piezoelectric systems use mechanical stress only as the initial stimulus. The key healing effect arises from stress‐induced polarization and the resulting local electric fields, which can regulate ion transport, homogenize interfacial flux, and improve reaction kinetics. Therefore, these strategies can be regarded as stress‐to‐electric‐field coupled self‐healing, bridging pressure/stress‐activated repair and electric‐field‐driven interfacial regulation. Such approach is particularly applicable to composite electrodes, where active materials continuously undergo volume changes and generate localized mechanical deformation during cycling. By converting this unavoidable mechanical deformation energy into localized polarization fields, piezoelectric materials can regulate interfacial reaction kinetics and alleviate stress concentration. This stress‐to‐electricity conversion endows the composite electrode interface with a spontaneous and continuous dynamic healing capability. Typical piezoelectric materials span organic polymers, inorganic ceramics, and their composite systems. Among them, inorganic piezoelectric ceramics and nanomaterials, such as lead zirconate titanate (PZT), barium titanate (BaTiO_3_), and zinc oxide (ZnO) possess exceptionally high piezoelectric coefficients, enabling them to generate strong polarization electric fields in response to minute mechanical stresses [177]. This exceptional stress‐responsive capability is particularly transformative for high‐capacity conversion cathode systems plagued by severe volume fluctuations [178]. Specifically, during charge/discharge cycles, the massive volume expansion of selenium or sulfur cathodes inevitably generates significant localized mechanical stress, particularly at the rigid sulfide SSE interface. However, when pre‐polarized PZT is integrated into the composite cathode, it acutely captures this destructive stress and translates it into a robust built‐in piezopotential. This dynamic, stress‐induced piezopotential alters the internal polarization intensity of the material, driving the continuous release of excess surface screening charges. These charges directly participate in the electrochemical reactions, thereby significantly accelerating the inherently sluggish conversion kinetics of polysulfides or polyselenides. Ultimately, this profound electro‐chemo‐mechanical coupling process in situ consumes the mechanical strain energy that would otherwise cause interfacial microcracks and delamination. By converting this destructive mechanical energy into beneficial electrochemical kinetic energy, it fundamentally dissipates the localized stress concentrations triggered by the accumulation of reaction intermediates and volumetric fluctuations. Similarly, in BaTiO_3_‐doped SSBs, this mechanism enables mechanical strain to induce a robust localized electric field. This self‐generated field effectively regulates the interfacial potential distribution and alleviates the space‐charge effect, ultimately yielding a more homogeneous ion flux and significantly enhanced interfacial stability (Figure 8f) [179].

Organic piezoelectric polymers with polar crystalline phases, typified by polyvinylidene fluoride (PVDF) and its copolymers such as PVDF‐HFP, exhibit exceptional flexibility and film‐forming processability [180]. By leveraging their intrinsic piezoelectric characteristics, these polymers can generate localized electric fields to homogenize ion deposition. Furthermore, uniformly dispersing highly active inorganic piezoelectric nanoparticles into such polymer matrices effectively synergizes the advantages of both organic and inorganic components. A ZnO/PVDF‐HFP self‐adaptation composite electrolyte was developed [181]. The organic‐inorganic synergistic piezoelectric effect can in situ convert the interfacial stress arising from volumetric fluctuations during Li plating/stripping into a local polarization electric field. This self‐generated field dynamically regulates the migration pathways and flux distribution of Li^+^, thereby lowering the local current density and fundamentally suppressing dendrite growth. Subsequently, piezoelectric gradient electrolytes have recently been developed by spatially tailoring the distribution of piezoelectric active components, forming a directional electric‐field gradient across the interfacial region [182]. This not only enables highly precise control over ion transport but also endows SSBs with an unprecedented adaptive response capability toward internal strain and environmental variations.

### 3.4 Internal Electric Field‐driven Self‐Healing

Electrochemically induced self‐healing goes beyond conventional externally applied thermal or stress stimulation. Instead, it uses the electrochemical field generated during charge/discharge as the driving force to induce real‐time interfacial structural evolution and dynamic phase transition [183]. Since local electric fields are widely distributed across different regions of the battery during cycling, this healing mode can be applied to various internal interfaces. Under this mechanism, specific battery components undergo controlled migration or redox reactions, forming low‐modulus, and highly flowable intermediate phases in situ. These intermediate phases continuously evolve in tandem with the fluctuating electric field. They adaptively adjust their spatial distribution in response to the volume fluctuation of active particles during lithiation/delithiation. The electric‐field‐driven phase transition or targeted mass migration constitutes the primary mechanism of this dynamic self‐healing behavior.

The Li_1.3_Fe_1.2_Cl_4_ halide, acting as an all‐in‐one cathode, exhibits a remarkable intrinsic self‐healing behavior during electrochemical cycling, arising from the dynamic interplay between its structural and mechanical responses [184]. Upon charging, Li^+^ extraction oxidizes from Fe^2+^ to Fe^3+^, accompanied by the reversible migration of local Fe ions from octahedral to tetrahedral sites (Figure 9a). This local Fe migration, coupled with multiphase transitions, enables structural rearrangement, and stress redistribution within defect‐prone regions, suppressing vacancy accumulation and crack propagation. Simultaneously, the material undergoes a brittle‐to‐ductile transition, in which the reduced melting point and enhanced plasticity promote interparticle fusion and local deformation (Figure 9b). These combined effects allow pre‐existing cracks and voids to close and self‐repair during charging. Experimental observations confirm that such defects are fully healed in the cycled electrode, resulting in a well‐connected, crack‐free microstructure that underpins exceptional cycling stability.

**FIGURE 9:**
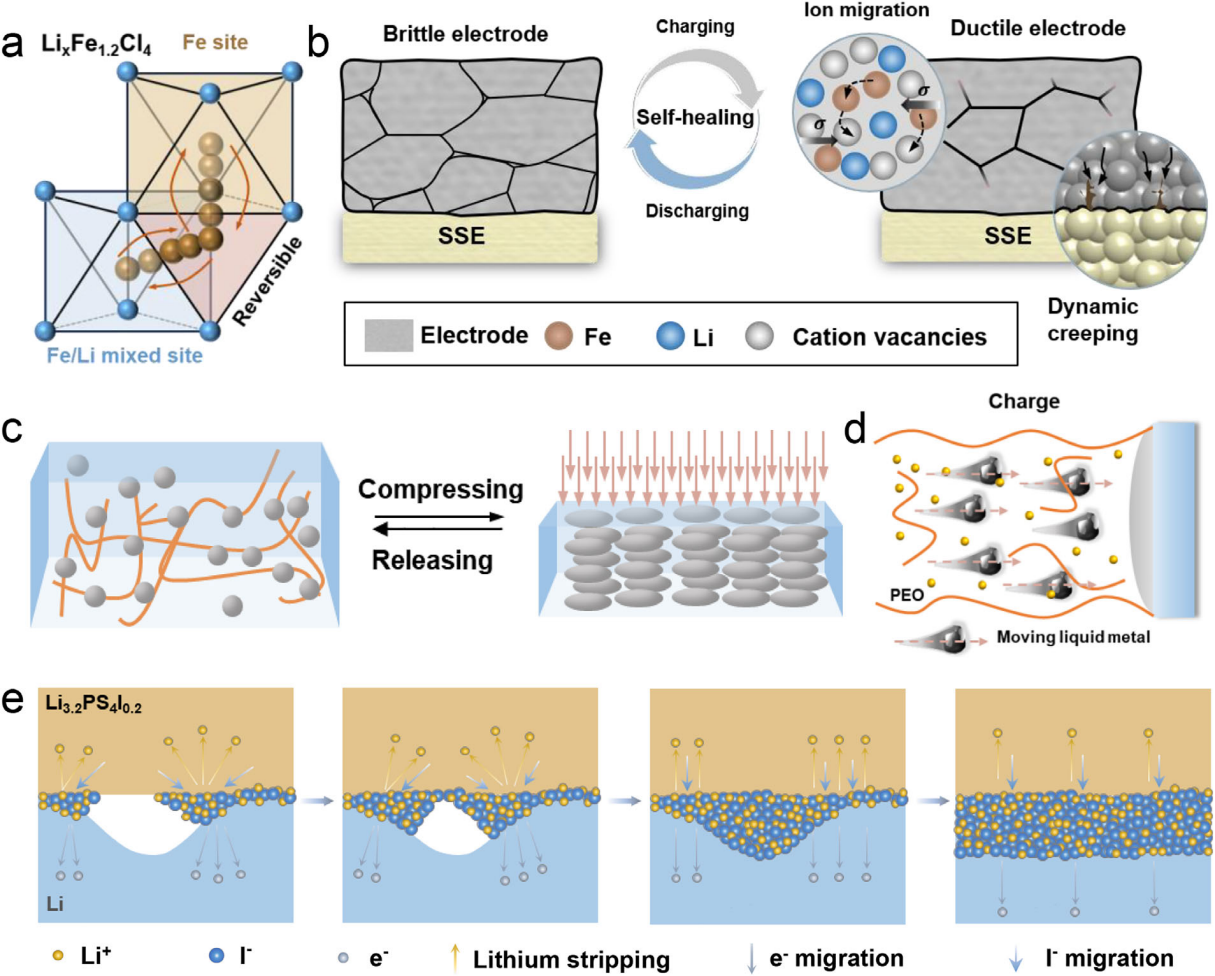
(a) The migration of iron between octahedral and tetrahedral sites in Li_x_Fe_1.2_Cl_4_. Adapted with permission [184]. Copyright 2025, Springer Nature. (b) The brittle‐ductile transition of the Li_x_Fe_1.2_Cl_4_ electrode during the charging and discharging process. Adapted with permission [184]. Copyright 2025, Springer Nature. (c) Deformation and repair of PEO‐based SSE containing GaInSn liquid metal under (de) pressurization. Adapted with permission [34]. Copyright 2024, Wiley. (d) Schematic diagram of the liquid metal moving towards the Na anode during the charging process. (e) The Li_3.2_PS_4_I_0.2_/Li interface evolution with I^–^ migration during stripping. Adapted with permission [185]. Copyright 2025, Springer Nature.

Under an internal electric field, strong electrostatic forces generate a directional migration vector for charged species. This driving force compels the adaptive redistribution of mobile ions, polar molecules, or flexible polymeric matrices toward interfacial defect regions. For instance, Suo et al. engineered a PEO‐based SSE incorporating GaInSn liquid‐metal nanoparticles [34]. When subjected to mechanical stress, the embedded liquid metal can dynamically deform to accommodate stress‐induced interfacial fluctuations and reversibly recover its shape once the stress is released (Figure 9c). Furthermore, driven by the electric field, the liquid metal flows to fill interfacial gaps, thereby restoring continuous electrode/electrolyte contact (Figure 9d). Concurrently, it facilitates the recovery of inactive Na and undergoes an alloying reaction with propagating dendrites, effectively arresting their growth while repairing existing interfacial defects.

The targeted migration of anions, particularly the electric‐field‐driven migration of halide anions toward the anode is common for alleviating anode/electrolyte interfacial degradation. Cen's group introduced mobile I^−^ into Li_3_PS_4_‐based SSE by doping LiI. During cycling, the I^−^ directionally migrated toward the Li/SSE interface and in situ formed a LiI‐rich dynamic adaptive interphase [185]. Unlike conventional preconstructed static interphases, this interphase continuously evolves with Li plating/stripping, enabling dynamic regulation on demand. Specifically, during Li stripping, when volume shrinkage of Li metal generates voids at the rigid SSE interface, the I^−^ accumulated at the interface promotes the preferential growth of the LiI phase within these defective regions (Figure 9e). In this way, local voids are dynamically filled, and interfacial contact is seamlessly reconstructed. Concurrently, this process reduces interfacial polarization, homogenizes the local current distribution, and effectively suppresses interfacial instability. A parallel anion‐migration mechanism has also been verified in Cl^−^ containing sulfide‐based SSBs. By tuning the lattice occupancy of Cl^−^ in the Li_6_PS_5_Cl electrolyte, Lu et al. found that part of the Cl^−^ can be released from the lattice and migrate to the Li anode, facilitating the in situ formation of LiCl‐rich interphase [186]. This locally enriched layer significantly enhances both the chemical stability and the physical contact compatibility of the Li/SSE interface. These results indicate that halide anion migration can not only regulate the interfacial chemical composition but also mitigate contact mismatch caused by interfacial morphological evolution through dynamic enrichment behavior.

Cation migration also induces adaptive interfacial reconstruction and shows self‐healing‐like behavior. Using in situ scanning electron microscopy (SEM), Zhao et al. revealed that during stripping of a Li‐rich Li‐Mg alloy anode, the interface avoids the continuous void accumulation and ultimate delamination typically observed in pure Li systems [94]. Instead, the boundary displays a distinctive splitting and collapse evolution pattern, which facilitates the partial recovery of physical contact. This unique behavior originates from the strong chemical affinity between the magnesium in the alloy and the sulfur species within the sulfide SSEs. This robust interaction propels magnesium migration toward the interface while concurrently driving Li vacancies into the bulk electrode, thereby suppressing the nucleation and coalescence of interfacial voids. Collectively, these findings indicate that the targeted migration of ions can not only dynamically regulate the interfacial chemical composition but also effectively mitigate contact mismatches induced by morphological evolution through an adaptive and electric‐field‐driven enrichment process.

### 3.5 Comparison of Different Self‐Healing Strategies

To provide a clearer comparison of different self‐healing strategies, representative systems and their reported healing‐related metrics are summarized in Table 1. Although the above self‐healing strategies are classified according to their repair mechanisms, their practical applicability is strongly interface‐dependent because cathode and anode interfaces operate under distinct electrochemical and chemical environments. At high‐voltage cathode interfaces, self‐healing components must tolerate oxidative potentials, reactive oxygen species, and possible interdiffusion between active materials and SSEs. Therefore, oxidation‐resistant polymer adhesives, piezoelectric or ferroelectric additives, and thermally responsive cathode‐active phases are generally more suitable for mitigating cathode‐side cracking and sluggish conversion reactions. In contrast, Li/Na metal and alloy anode interfaces are dominated by low redox potentials, stripping‐induced voids, and dendrite penetration. Flowable metals, soft alloy phases, pressure‐assisted creep, and ion‐migration‐induced adaptive interphases are therefore more effective for restoring contact and homogenizing ion flux at the anode side, provided that they form chemically stable and electronically insulating interphases. For Si‐based anodes, strategies combining elastic accommodation with chemical compatibility, such as dynamic polymer networks and liquid‐metal‐assisted conductive networks, are particularly useful for maintaining ion/electron pathways during large volume changes.

**TABLE 1:** Comparison of representative interfacial self‐healing strategies and reported healing‐related metrics in SSBs.

Healing strategies	Target interface	Resistance recovery	Capacity retention after healing	Number of healing cycles	Refs.
Viscoelastic polymers	Cathode & anode/electrolyte interface	N/A	88% (5 mA cm^−2^, 1100 cycles)	Continuous (in situ)	[149]
Low‐melting‐point alloys and metals	µSi anode/electrolyte interface	Reduced by 72.4%	98.4% (1 C, 200 cycles)	Continuous (in situ)	[156]
Reversible covalent bonding	Li anode/electrolyte interface	Reduced by 46.6%	74.9% (0.1 C, 100 cycles)	Continuous (in situ)	[166]
Supramolecular interaction	Li anode/electrolyte interface	N/A	91.2% (0.5 C, 206 cycles)	Continuous (in situ)	[172]
Thermally activated self‐healing	S_9.3_I cathode/electrolyte interface	N/A	87% (0.16 A g^−1^, 400)	8 times	[175]
Pressure‐activated self‐healing	Li anode/electrolyte interface	N/A	96% (0.5 C, 400 cycles)	Continuous (in situ)	[181]
Internal electric field‐driven self‐healing	Li anode/electrolyte interface	N/A	90.7% (2 C, 2400 cycles)	Continuous (in situ)	[186]

## 4 Summary and Outlook

SSBs are widely regarded as one of the most promising next‐generation energy storage systems due to their intrinsic safety and potential for high energy density. However, the current cyclic life and fast charging capability remain insufficient to meet the demands of practical, large‐scale commercial applications. This fundamental bottleneck largely stems from the instability of solid‐solid interfaces. Therefore, a comprehensive understanding and in‐depth exploration of the intertwined electro‐chemo‐mechanical degradation mechanisms are of paramount importance for designing intrinsically stable interfaces. This review has systematically analyzed the multifaceted interfacial degradation pathways, with a specific focus on mechanical structural failures, parasitic electro(chemical) side reactions, and the severe detrimental coupling behaviors between them. By elucidating these failure modes, we propose that developing dynamic interfacial self‐healing strategies represents a highly viable and necessary approach to resolving these interfacial dilemmas. Unlike conventional static protective layers, which are prone to secondary failure under continuous stress, dynamic self‐healing systems can spontaneously reconstruct broken interfaces and adaptively conform to the severe volume fluctuations of active materials. By integrating dynamic physical and chemical interactions or multi‐field coupled smart responsive mechanisms, these self‐healing architectures provide unprecedented pathways to actively relieve interfacial stress, suppress parasitic side reactions, and maintain stable Li^+^ transport. However, despite the demonstrable effectiveness of current dynamic self‐healing strategies in mitigating interfacial degradation, several critical challenges and potential issues remain to be addressed in future research:

1. Ideally, self‐healing capability should be an intrinsic property of the fundamental battery components. A crucial prerequisite in designing such dynamic self‐healing interfaces is to avoid introducing secondary defects that could further accelerate capacity degradation. If incorporating external self‐healing materials (such as specifically functionalized polymers) is unavoidable, it is imperative that they neither impede charge carrier transport nor operate outside the electrochemical stability window of the cell. For instance, polymer‐based self‐healing systems, particularly those relying on dynamic covalent bonds like disulfide or imine linkages, are highly susceptible to severe oxidation at the high‐voltage cathode interface. Therefore, lowering the highest occupied molecular orbital (HOMO) energy level of these polymers to thermodynamically enhance their oxidation resistance is an indispensable direction for future research.
2. Direct visualization and real‐time tracking of interfacial healing processes remain highly important. Future studies should establish mechanism‐specific operando characterization protocols to directly verify interfacial self‐healing in SSBs. For physical flow and space‐filling strategies, operando optical microscopy, SEM, or x‐ray tomography combined with EIS can be used to track crack closure, void filling, and interfacial resistance reduction. For dynamic bonding or supramolecular healing, in situ spectroscopic techniques, such as FTIR or Raman, together with mechanical/electrochemical monitoring, can reveal reversible bond reconstruction and stress relaxation. For thermally or pressure‐triggered healing, operando heating/pressure cells coupled with imaging and impedance analysis can clarify whether phase transition, softening, or creep deformation truly restores interfacial contact. For electric‐field‐driven or ion‐migration‐induced healing, operando Raman, quasi in situ XPS, and EIS can identify adaptive interphase formation and ion‐flux homogenization. Such multimodal evidence would help distinguish true self‐healing behavior from simple performance stabilization and provide quantitative descriptors.
3. The trade‐off between dynamic repair efficiency and mechanical robustness remains critical. A quantitative evaluation of healing efficiency is essential for assessing this balance. For interfacial transport degradation, resistance recovery measured by in situ EIS can serve as a direct indicator, while capacity recovery after healing is more relevant to practical cell performance. However, capacity recovery should be correlated with morphological or impedance evidence to avoid confusing true self‐healing with simple kinetic enhancement. In general, efficient repair requires low modulus and high chain mobility, which facilitate crack closure, void filling, and interfacial reconstruction. However, overly soft matrices may provide insufficient mechanical support, leaving the interface vulnerable to Li dendrite penetration and irreversible deformation under prolonged stack pressure or cycling stress. Multi‐phase designs that combine rigid supports, such as high‐modulus inorganic frameworks or permanent covalent networks, with dynamic repair phases can help mitigate this conflict. Further optimization of interphase matching, stress transfer, and energy dissipation is therefore needed to achieve both high repair efficiency and mechanical integrity.
4. Efficient self‐healing demands that materials maintain long‐term stability and sustainable responsiveness under complex operating conditions. Interfacial degradation inherently involves intricate coupled effects, encompassing mechanical stress, electrochemical side reactions, and space charge accumulation. However, most current self‐healing designs predominantly focus on mitigating reversible physical defects, paying limited attention to irreversible chemical degradation or the fatigue of dynamic functional groups over prolonged cycling. Future strategies can synergize dynamic self‐healing mechanisms with static performance enhancement approaches to facilitate comprehensive, multi‐field (electro‐chemo‐mechanical) repair. By addressing these coupled degradation pathways simultaneously, integrated designs can effectively alleviate the operational stress on the battery and extend its cycle life.
5. From an industrial perspective, the practical implementation of self‐healing strategies in solid‐state batteries will likely rely on built‐in adaptive components rather than post‐failure repair. One feasible route is to incorporate self‐healing polymers as binders, adhesive interlayers, or composite electrolyte components through scalable electrode coating or dry‐processing methods. These components can buffer stress, maintain solid‐solid contact, and repair local interfacial defects during cycling, provided that their redox stability and transport properties are compatible with the target interface. Another promising approach is to construct adaptive anode interphases through soft alloy layers, low‐modulus metal components, or formation‐induced ion migration, which can mitigate stripping‐induced voids and regulate Li/Na deposition. In addition, thermally responsive materials and piezoelectric additives may be integrated with battery thermal or mechanical management systems to enable on‐demand interfacial reconstruction.

## Author Contributions

**Xinxin Zhu**: Writing – original draft, conceptualization, methodology. **Tengfei Dai**: Data curation. **Wendi Dou**: Data curation. **Zhiheng Li**: Visualization. **Kaihua Wen**: Writing – review and editing. **Yanqun Lv**: Methodology. **Ming Zhao**: Conceptualization. **Jun Lu**: Writing – review and editing, investigation, funding acquisition. **Zaiping Guo**: Writing – review and editing, funding acquisition, conceptualization, investigation, validation.

## Conflicts of Interest

The authors declare no conflicts of interest.

## Data Availability

The data that support the findings of this study are available from the corresponding author upon reasonable request.

## References

[1] R. Chen , Q. Li , X. Yu , L. Chen , and H. Li , “Approaching Practically Accessible Solid‐State Batteries: Stability Issues Related to Solid Electrolytes and Interfaces,” Chemical Reviews 120 (2020): 6820–6877, 10.1021/acs.chemrev.9b00268. 31763824

[2] J. Janek and W. G. Zeier , “Challenges in Speeding up Solid‐State Battery Development,” Nature Energy 8 (2023): 230–240, 10.1038/s41560-023-01208-9.

[3] W. Jiang , L. Yan , X. Zeng , et al., “Adhesive Sulfide Solid Electrolyte Interface for Lithium Metal Batteries,” ACS Applied Materials & Interfaces 12 (2020): 54876–54883, 10.1021/acsami.0c17828. 33236875

[4] X. Miao , S. Guan , C. Ma , L. Li , and C.‐W. Nan , “Role of Interfaces in Solid‐State Batteries,” Advanced Materials 35 (2023): 2206402, 10.1002/adma.202206402. 36062873

[5] E. P. Alsaç , D. L. Nelson , S. G. Yoon , et al., “Characterizing Electrode Materials and Interfaces in Solid‐State Batteries,” Chemical Reviews 125 (2025): 2009–2119, 10.1021/acs.chemrev.4c00584. PMC11869192 39903474

[6] X. Yu , R. Chen , L. Gan , H. Li , and L. Chen , “Battery Safety: From Lithium‐Ion to Solid‐State Batteries,” Engineering 21 (2023): 9–14, 10.1016/j.eng.2022.06.022.

[7] J. Zhang , J. Fu , P. Lu , et al., “Challenges and Strategies of Low‐Pressure all‐Solid‐State Batteries,” Advanced Materials 37 (2025): 2413499, 10.1002/adma.202413499. PMC11817941 39726082

[8] F. Zhang , Y. Guo , L. Zhang , et al., “A Review of the Effect of External Pressure on all‐Solid‐State Batteries,” eTransportation 15 (2023): 100220, 10.1016/j.etran.2022.100220.

[9] J.‐M. Doux , H. Nguyen , D. H. S. Tan , et al., “Stack Pressure Considerations for Room‐Temperature all‐Solid‐State Lithium Metal Batteries,” Advanced Energy Materials 10 (2020): 1903253, 10.1002/aenm.201903253.

[10] X. Zhu , W. Jiang , S. Zhao , et al., “Exploring the Concordant Solid‐State Electrolytes for all‐Solid‐State Lithium‐Sulfur Batteries,” Nano Energy 96 (2022): 107093, 10.1016/j.nanoen.2022.107093.

[11] L. Wang , B. Hu , C. Doerrer , et al., “Understanding the Role of Triple Phase Boundaries on Coating‐Free Solid‐State Cathodes,” ACS Energy Letters 11 (2026): 1623–1630, 10.1021/acsenergylett.5c02607. PMC12910715 41710784

[12] W. Xia , Y. Zhao , F. Zhao , et al., “Antiperovskite Electrolytes for Solid‐State Batteries,” Chemical Reviews 122 (2022): 3763–3819, 10.1021/acs.chemrev.1c00594. 35015520

[13] K. H. Baeck , Y. B. Song , D. Kim , et al., “Chemo‐Mechanical Failure and Reinforcement of Solid Electrolyte Films for Practical All‐Solid‐State Li Metal Pouch Cells,” Advanced Materials 38 (2026): 18655, 10.1002/adma.202518655. 41706542

[14] A. De Gol , K. B. Dermenci , L. Farkas , and M. Berecibar , “Electro‐Chemo‐Mechanical Degradation in Solid‐State Batteries: A Review of Microscale and Multiphysics Modeling,” Advanced Energy Materials 14 (2024): 2403255, 10.1002/aenm.202403255.

[15] W. Jiang , X. Zhu , Y. Liu , et al., “Mechanically Reinforced Ni‐Rich Cathodes for High‐Power and Long‐Life All‐Solid‐State Batteries,” Chemical Engineering Science 288 (2024): 119775, 10.1016/j.ces.2024.119775.

[16] X. Bai , F. Xie , Z. Zhang , et al., “Prospects and Strategies for Single‐Crystal NCM Materials to Solve all‐Solid‐State Battery Cathode Interface Problems,” Advanced Energy Materials 14 (2024): 2401336, 10.1002/aenm.202401336.

[17] R. S. Negi , Y. Yusim , R. Pan , et al., “A Dry‐Processed Al_2_O_3_/LiAlO_2_ Coating for Stabilizing the Cathode/Electrolyte Interface in High‐Ni Ncm‐Based All‐Solid‐State Batteries,” Advanced Materials 9 (2022): 2101428, 10.1002/admi.202101428.

[18] X. Zhu , J. Wu , and J. Lu , “Insight Into Inorganic Solid‐State Electrolytes: Ionic Transport and Failure Mechanisms,” Advanced Functional Materials 34 (2024): 2409547, 10.1002/adfm.202409547.

[19] Y. Wang , S. Zhang , H. Hua , et al., “Recent Advances in the Failure Analysis of Solid‐State Li Ion Batteries,” Renewables 3 (2025): 175–202, 10.31635/renewables.025.202500090.

[20] J. Wang , Z. Zhang , J. Han , et al., “Interfacial and Cycle Stability of Sulfide All‐Solid‐State Batteries With Ni‐Rich Layered Oxide Cathodes,” Nano Energy 100 (2022): 107528, 10.1016/j.nanoen.2022.107528.

[21] Q. Zhao , S. Stalin , C. Zhao , and L. A. Archer , “Designing Solid‐State Electrolytes for Safe, Energy‐Dense Batteries,” Nature Reviews Materials 5 (2020): 229–252, 10.1038/s41578-019-0165-5.

[22] L. Ye and X. Li , “A Dynamic Stability Design Strategy for Lithium Metal Solid State Batteries,” Nature 593 (2021): 218–222, 10.1038/s41586-021-03486-3. 33981053

[23] K. B. Hatzell and L. F. Nazar , “Recent Advances in Solid‐State Batteries,” Journal of the American Chemical Society 147 (2025): 14881–14883, 10.1021/jacs.5c06058. 42704789

[24] J. Yu , X. Sun , X. Shen , et al., “Stack Pressure‐a Critical Strategy and Challenge in Performance Optimization of Solid State Batteries,” Energy Storage Materials 76 (2025): 104134, 10.1016/j.ensm.2025.104134.

[25] H. Huo , Y. Bai , S. L. Benz , et al., “Decoupling the Effects of Interface Chemical Degradation and Mechanical Cracking in Solid‐State Batteries With Silicon Electrode,” Advanced Materials 37 (2025): 2415006, 10.1002/adma.202415006. PMC11837880 39703118

[26] X. Zhu , W. Jiang , L. Wang , and J. Lu , “Constructing Resilient Cross‐Linked Network Toward Stable All‐Solid‐State Lithium‐Sulfur Batteries,” Advanced Energy Materials 14 (2024), 2304244, 10.1002/aenm.202304244.

[27] Z. Liang , Y. Xiang , K. Wang , et al., “Understanding the Failure Process of Sulfide‐Based all‐Solid‐State Lithium Batteries via Operando Nuclear Magnetic Resonance Spectroscopy,” Nature Communications 14 (2023): 259, 10.1038/s41467-023-35920-7. PMC9845218 36650152

[28] G. Zhao , W. Liu , B. Zhang , et al., “Zr_2_O_12_‐Lithium Interphase for High Performance Solid Lithium Metal Batteries,” Renewables 3 (2025): 21–30, 10.31635/renewables.025.202400075.

[29] C. Wang , “Towards Practical All‐Solid‐State Batteries,” Nature Sustainability 8 (2025): 1246–1247, 10.1038/s41893-025-01621-w.

[30] S. Lee , T. Kim , K. Kim , et al., “Mechano‐Electrochemical Healing at the Interphase between LiNi_0.8_Co_0.1_Mn_0.1_O_2_ and Li_6_PS_5_Cl in all‐Solid‐State Batteries,” Advanced Energy Materials 15 (2025): 2405782, 10.1002/aenm.202405782.

[31] X. Wang , S. He , Z. Hu , H. Xu , L. Pan , and J. Li , “Grafting Strategy Achieving Self‐Healing Polymer/Sulfide Electrolyte for High‐Performance Solid‐state Lithium–silicon Batteries,” Rare Metals 44 (2025): 7159–7172, 10.1007/s12598-025-03412-w.

[32] D. Wu , Z. Zhang , L. Wang , et al., “Self‐Adaptive Interfacial Glue for Low‐Pressure Sulfide‐Based All‐Solid‐State Lithium Metal Batteries,” Energy & Environmental Science 19 (2026): 1630–1641, 10.1039/d5ee06207a.

[33] V.‐P. Vu , H.‐M. So , A. Kim , J. Y. Lee , M. Oh , and S. Hyun , “Self‐Healing Polymer Binders: Next‐Generation Battery Applications,” Journal of Materials Chemistry A 13 (2025): 38541–38571, 10.1039/D5TA04403K.

[34] J. Suo , Y. Jia , X. Zhu , et al., “A Novel Super‐Toughness and Self‐Healing Solid Polymer Electrolyte for Solid Sodium Metal Batteries,” Advanced Materials 36 (2024): 2409587, 10.1002/adma.202409587. 39396372

[35] B. Fang , L. Wang , Z. Wang , Z. Su , F. Zou , and R. Mo , “Self‐Healing Interfacial Cross‐Links Enable Supertough Solid Polymer Electrolytes With Eliminating‐Dendrite Lithium Metal Battery,” ACS Materials Letters 7 (2025): 3292–3300, 10.1021/acsmaterialslett.5c01098.

[36] Y. Jiang , K. Chen , J. He , et al., “A Self‐Healing Composite Solid Electrolyte With Dynamic Three‐Dimensional Inorganic/Organic Hybrid Network for Flexible All‐Solid‐State Lithium Metal Batteries,” Journal of Colloid and Interface Science 678 (2025): 200–209, 10.1016/j.jcis.2024.09.119. 39293364

[37] T. Yang , S. Qin , S. Gao , et al., “Electroinitiated Interfacial Healing for External Pressure‐Free Solid‐State Sodium Metal Batteries,” Nature Communications 16 (2025): 9613, 10.1038/s41467-025-64612-7. PMC12575646 41168187

[38] J. Zheng , X. Zhu , L. Wang , J. Lu , and T. Wu , “Insights Into Interfacial Physiochemistry in Sulfide Solid‐State Batteries: A Review,” Materials Chemistry Frontiers 7 (2023): 4810–4832, 10.1039/d3qm00400g.

[39] L. Xi , D. Zhang , X. Xu , et al., “Interface Engineering of all‐Solid‐State Batteries Based on Inorganic Solid Electrolytes,” Chemistry‐Sustainability‐Energy‐Materials 16 (2023): 202202158, 10.1002/cssc.202202158. 36658096

[40] Z. Deng , S. Chen , K. Yang , et al., “Tailoring Interfacial Structures to Regulate Carrier Transport in Solid‐State Batteries,” Advanced Materials 36 (2024): 2407923, 10.1002/adma.202407923. 39081109

[41] P. He , Y. Tang , Z. Tan , et al., “Solid‐State Batteries Encounter Challenges Regarding the Interface Involving Lithium Metal,” Nano Energy 124 (2024): 109502, 10.1016/j.nanoen.2024.109502.

[42] A. C. C. Dutra , B. A. Goldmann , M. S. Islam , and J. A. Dawson , “Understanding Solid‐State Battery Electrolytes Using Atomistic Modelling and Machine Learning,” Nature Reviews Materials 10 (2025): 566–583, 10.1038/s41578-025-00817-y.

[43] J. A. Lewis , J. Tippens , F. J. Q. Cortes , and M. T. McDowell , “Chemo‐Mechanical Challenges in Solid‐State Batteries,” Trends in Chemistry 1 (2019): 845–857, 10.1016/j.trechm.2019.06.013.

[44] C. Yuan , J. Wu , W. Zhang , M. Han , and Y. Jia , “Electro‐Chemo‐Mechanics Interplays Caused by Solid Electrolyte‐Lithium Anode Interface Roughness in all‐Solid‐State Batteries,” Journal of Energy Chemistry 108 (2025): 495–507, 10.1016/j.jechem.2025.04.033.

[45] Y. Zhong , X. Zhang , Y. Zhang , et al., “Understanding and Unveiling the Electro‐Chemo‐Mechanical Behavior in Solid‐State Batteries,” Sustainable Materials 4 (2024), e190, 10.1002/sus2.190.

[46] S. Kalnaus , N. J. Dudney , A. S. Westover , E. Herbert , and S. Hackney , “Solid‐State Batteries: The Critical Role of Mechanics,” Science 381 (2023): abg5998, 10.1126/science.abg5998. 37733866

[47] J. Gu , X. Chen , R. Ma , et al., “The Insight of Micro‐Short Circuits Caused by Electrochemo‐Mechanical Stress Crosstalk in all‐Solid‐State Li Metal Batteries,” Energy Storage Materials 63 (2023): 103052, 10.1016/j.ensm.2023.103052.

[48] J. Gu , Z. Liang , J. Shi , and Y. Yang , “Electrochemo‐Mechanical Stresses and Their Measurements in Sulfide‐Based all‐Solid‐State Batteries: A Review,” Advanced Energy Materials 13 (2023): 2203153, 10.1002/aenm.202203153.

[49] X. Zhu , L. Wang , Z. Bai , J. Lu , and T. Wu , “Sulfide‐Based all‐Solid‐State Lithium–Sulfur Batteries: Challenges and Perspectives,” Nano‐Micro Letters 15 (2023): 75, 10.1007/s40820-023-01053-1. PMC10050614 36976391

[50] W. Jiang , X. Fan , X. Zhu , et al., “Well‐Dispersed Single‐Crystalline Nickel‐Rich Cathode for Long‐Life High‐Voltage All‐Solid‐State Batteries,” Journal of Power Sources 508 (2021): 230335, 10.1016/j.jpowsour.2021.230335.

[51] S. Dong , L. Sheng , L. Wang , et al., “Challenges and Prospects of all‐Solid‐State Electrodes for Solid‐State Lithium Batteries,” Advanced Functional Materials 33 (2023): 2304371, 10.1002/adfm.202304371.

[52] Q. Wang , D. Zhou , C. Zhao , et al., “Fast‐Charge High‐Voltage Layered Cathodes for Sodium‐Ion Batteries,” Nature Sustainability 7 (2024): 338–347, 10.1038/s41893-024-01266-1.

[53] X. Zhu , W. Wang , W. Dou , et al., “Directional Catalysis of Sulfur at Highly Ordered Triple‐Phase Interfaces in all‐Solid‐State Lithium‐Sulfur Batteries,” Advanced Materials 38 (2026): 22483, 10.1002/adma.202522483. 41630139

[54] L.‐Z. Fan , H. He , and C.‐W. Nan , “Tailoring Inorganic–Polymer Composites for the Mass Production of Solid‐State Batteries,” Nature Reviews Materials 6 (2021): 1003–1019, 10.1038/s41578-021-00320-0.

[55] G. Bucci , T. Swamy , Y.‐M. Chiang , and W. C. Carter , “Modeling of Internal Mechanical Failure of all‐Solid‐State Batteries during Electrochemical Cycling, and Implications for Battery Design,” Journal of Materials Chemistry A 5 (2017): 19422–19430, 10.1039/C7TA03199H.

[56] H. Xu , S. Yang , and B. Li , “Pressure Effects and Countermeasures in Solid‐State Batteries: A Comprehensive Review,” Advanced Energy Materials 14 (2024): 2303539, 10.1002/aenm.202303539.

[57] H.‐S. Zhang , X.‐C. Lei , D. Su , et al., “Surface Lattice Modulation Enables Stable Cycling of High‐loading all‐Solid‐state Batteries at High Voltages,” Angewandte Chemie International Edition 63 (2024): 202400562, 10.1002/anie.202400562. 38382041

[58] Y. Gong , Y. Chen , Q. Zhang , et al., “Three‐Dimensional Atomic‐Scale Observation of Structural Evolution of Cathode Material in a Working all‐Solid‐State Battery,” Nature Communications 9 (2018): 3341, 10.1038/s41467-018-05833-x. PMC6104093 30131492

[59] H.‐H. Ryu , B. Namkoong , J.‐H. Kim , I. Belharouak , C. S. Yoon , and Y.‐K. Sun , “Capacity Fading Mechanisms in Ni‐Rich Single‐Crystal Ncm Cathodes,” ACS Energy Letters 6 (2021): 2726–2734, 10.1021/acsenergylett.1c01089.

[60] C. Wang , Y. Zhang , Z. Li , et al., “A Comprehensive Understanding of the Structural Evolution and Capacity Contribution of Fast‐Charging Ncm Cathodes,” Journal of Energy Chemistry 114 (2026): 183–193, 10.1016/j.jechem.2025.09.066.

[61] J. Li , Y. He , S. Nazari , et al., “Revealing the Degradation Behaviors and Mechanisms of Ncm Cathode in Scrapped Lithium‐Ion Batteries,” Journal of Power Sources 582 (2023): 233563, 10.1016/j.jpowsour.2023.233563.

[62] J. Zuo , L. Yang , X. Yang , et al., “Reconstruction of R 3 m Disordered Phase Homogenizing Li ^+^ Distribution toward Mitigating H2‐H3 Phase Transition in LiNi_0.95_Co_0.02_Mn_0.03_O_2_ ,” Advanced Materials 38 (2026): 23158, 10.1002/adma.202523158. 41803076

[63] M. Gong , Y. Wei , H. Zhang , et al., “High‐Entropy Doped Ni‐Rich Oxide Cathodes With Alleviated H2–H3 Phase Transition for Li‐Ion and All‐Solid‐State Li Batteries,” ACS Sustainable Chemistry & Engineering 13 (2025): 10831–10841, 10.1021/acssuschemeng.5c01651.

[64] J. Guo , P. Li , F. Piccolo , et al., “Degradation and Failure Mechanisms of Lithium/LiNi_x_Co_y_Mn_1–X–Y_O_2_ Batteries,” ACS Energy Letters 10 (2025): 2318–2340, 10.1021/acsenergylett.4c03578.

[65] G. Zhao , Y. Sun , H. Ma , et al., “Exploring Degradation Mechanisms and Recent Developments in High‐Nickel Layered Cathodes for Lithium Batteries,” Electrochemical Energy Reviews 8 (2025): 21, 10.1007/s41918-025-00254-z.

[66] Y. Zou , D. Yu , Y. Tang , et al., “The Dilemma of Single‐crystal High‐nickel LiNi_x_Co_y_Mn_1‐x‐y_O_2_ (x ≥ 0.9) Cathodes: Inhomogeneous Delithiation inside and outside the Particle,” Chemical Engineering Journal 504 (2025): 158800, 10.1016/j.cej.2024.158800.

[67] G.‐T. Park , H.‐H. Ryu , N.‐Y. Park , S.‐B. Lee , and Y.‐K. Sun , “Single‐Crystal vs Polycrystalline Cathodes for Lithium‐Ion Batteries,” Chemical Reviews 125 (2025): 9930–10000, 10.1021/acs.chemrev.5c00441. 40977510

[68] X. Kong , Y. Zhang , S. Peng , J. Zeng , and J. Zhao , “Superiority of Single‐Crystal to Polycrystalline LiNi_x_Co_y_Mn_1–X–Y_O_2_ Cathode Materials in Storage Behaviors for Lithium‐Ion Batteries,” ACS Sustainable Chemistry & Engineering 8 (2020): 14938–14948, 10.1021/acssuschemeng.0c05011.

[69] L. Xu , S. Tang , Y. Cheng , et al., “Interfaces in Solid‐State Lithium Batteries,” Joule 2 (2018): 1991–2015, 10.1016/j.joule.2018.07.009.

[70] P. Barai , T. Rojas , B. Narayanan , A. T. Ngo , L. A. Curtiss , and V. Srinivasan , “Investigation of Delamination‐Induced Performance Decay at the Cathode/LLZO Interface,” Chemistry of Materials 33 (2021): 5527–5541, 10.1021/acs.chemmater.0c04656.

[71] Z. Deng , Z. Wang , I.‐H. Chu , J. Luo , and S. P. Ong , “Elastic Properties of Alkali Superionic Conductor Electrolytes From First Principles Calculations,” Journal of The Electrochemical Society 163 (2016): A67–A74, 10.1149/2.0061602jes.

[72] J. Ji , B. Li , and W.‐H. Zhong , “Effects of a Block Copolymer as Multifunctional Fillers on Ionic Conductivity, Mechanical Properties, and Dimensional Stability of Solid Polymer Electrolytes,” The Journal of Physical Chemistry B 114 (2010): 13637–13643, 10.1021/jp105816s. 20936864

[73] C. Yuan , J. Wu , W. Zhang , and J. Xu , “Decoding Electrochemo‐Mechanical Degradation in Solid‐State Batteries: A Phase‐Field Study Toward Robust Cathode Design,” Energy Storage Materials 82 (2025): 104577, 10.1016/j.ensm.2025.104577.

[74] S. Moradi , B. Zahiri , and P. V. Braun , “Cathode Chemomechanics Controls Li Metal Solid‐State Battery Performance Under Low Stack Pressures,” Nature Communications 16 (2025): 9266, 10.1038/s41467-025-64358-2. PMC12537972 41115896

[75] G. Pustorino , H. Jagad , W. Li , et al., “Mechanical and Electronic Properties of Bulk and Surface Li_6_PS_5_Cl Argyrodite: First‐Principles Insights on Li‐Filament Resistance,” Chemistry of Materials 37 (2025): 313–321, 10.1021/acs.chemmater.4c02577.

[76] J. Gu , X. Chen , Z. He , et al., “Decoding Internal Stress‐Induced Micro‐Short Circuit Events in Sulfide‐Based all‐Solid‐State Li‐Metal Batteries via Operando Pressure Measurements,” Advanced Energy Materials 13 (2023): 2302643, 10.1002/aenm.202302643.

[77] Z. Fu and E. Wachsman , “Mechanical Properties of Three‐Dimensional Trilayered Li–garnet Electrolyte for High‐Rate Cycling in Solid‐state Batteries,” Journal of the American Ceramic Society 107 (2024): 1481–1489, 10.1111/jace.19339.

[78] H. Pan , M. Zhang , Z. Cheng , et al., “Carbon‐Free and Binder‐Free Li‐Al Alloy Anode Enabling an All‐Solid‐State Li‐S Battery With High Energy and Stability,” Science Advances 8 (2022): abn4372, 10.1126/sciadv.abn4372. PMC9007512 35417237

[79] D. Wang , L.‐J. Jhang , R. Kou , et al., “Realizing High‐Capacity all‐Solid‐State Lithium‐Sulfur Batteries Using a Low‐Density Inorganic Solid‐State Electrolyte,” Nature Communications 14 (2023): 1895, 10.1038/s41467-023-37564-z. PMC10076334 37019929

[80] H. Li , Y. Li , and L. Zhang , “Designing Principles of Advanced Sulfur Cathodes Toward Practical Lithium‐Sulfur Batteries,” Sustainable Materials 2 (2022): 34–64, 10.1002/sus2.42.

[81] Y. S. Oh , M. Kim , S. Kang , J.‐Y. Park , and H.‐T. Lim , “Redox Activity of Li_2_S–P_2_S_5_ Electrolyte Inducing Chemo‐Mechanical Failure in all‐Solid‐State Batteries Comprising Sulfur Composite Cathode and Li–Si Alloy Anode,” Chemical Engineering Journal 442 (2022): 136229, 10.1016/j.cej.2022.136229.

[82] Y. Zhou , D. Chen , X. Ren , et al., “Reliable Sulfur Cathode Design for all‐Solid‐State Lithium Metal Batteries Based on Sulfide Electrolytes,” Advanced Energy Materials 15 (2025): 2500061, 10.1002/aenm.202500061.

[83] D. Wang , B. Gwalani , D. Wierzbicki , et al., “Overcoming the Conversion Reaction Limitation at Three‐Phase Interfaces Using Mixed Conductors towards Energy‐Dense Solid‐State Li–S Batteries,” Nature Materials 24 (2025): 243–251, 10.1038/s41563-024-02057-x. 39762497

[84] J. Xiao , Q. Li , Y. Bi , et al., “Understanding and Applying Coulombic Efficiency in Lithium Metal Batteries,” Nature Energy 5 (2020): 561–568, 10.1038/s41560-020-0648-z.

[85] C. Fang , J. Li , M. Zhang , et al., “Quantifying Inactive Lithium in Lithium Metal Batteries,” Nature 572 (2019): 511–515, 10.1038/s41586-019-1481-z. 31435056

[86] L. Fu , M. Wan , B. Zhang , et al., “A Lithium Metal Anode Surviving Battery Cycling Above 200°C,” Advanced Materials 32 (2020): 2000952, 10.1002/adma.202000952. 32468648

[87] Y. Hu , Z. Chen , Y. Wang , et al., “Review of Thin Lithium Metal Battery Anode Fabrication—Microstructure—Electrochemistry Relations,” Advanced Materials 38 (2026): 11817, 10.1002/adma.202511817. PMC12933024 41078014

[88] J. Liu , H. Yuan , H. Liu , et al., “Unlocking the Failure Mechanism of Solid State Lithium Metal Batteries,” Advanced Energy Materials 12 (2022): 2100748, 10.1002/aenm.202100748.

[89] R.‐M. Gao , H. Yang , C.‐Y. Wang , H. Ye , F.‐F. Cao , and Z.‐P. Guo , “Fatigue‐Resistant Interfacial Layer for Safe Lithium Metal Batteries,” Angewandte Chemie 133 (2021): 25712–25717, 10.1002/ange.202111199. 34580988

[90] T. Wang , B. Chen , Y. Liu , et al., “Fatigue of Li Metal Anode in Solid‐State Batteries,” Science 388 (2025): 311–316, 10.1126/science.adq6807. 40245125

[91] Y. Moon , H. Kim , M. H. Nguyen , C. K. Song , W.‐J. Song , and S. Park , “Harnessing Native Li_2_CO_3_ Layers on Li_7_La_3_Zr_2_O_12_ (LLZO) for High‐Performance Tri‐Layer Composite Electrolytes in Semi‐Solid‐State Batteries,” Small 21 (2025): 09544, 10.1002/smll.202509544. 41170759

[92] C. D. Fincher , C. E. Athanasiou , C. Gilgenbach , et al., “Controlling Dendrite Propagation in Solid‐State Batteries With Engineered Stress,” Joule 6 (2022): 2794–2809, 10.1016/j.joule.2022.10.011.

[93] Y. Chen , Z. Wang , X. Li , et al., “Li Metal Deposition and Stripping in a Solid‐State Battery via Coble Creep,” Nature 578 (2020): 251–255, 10.1038/s41586-020-1972-y. 32015545

[94] L. Zhao , M. Feng , C. Wu , et al., “Imaging the Evolution of Lithium‐Solid Electrolyte Interface Using Operando Scanning Electron Microscopy,” Nature Communications 16 (2025): 4283, 10.1038/s41467-025-59567-8. PMC12062494 40341561

[95] L. Albero Blanquer , F. Marchini , J. R. Seitz , et al., “Optical Sensors for Operando Stress Monitoring in Lithium‐Based Batteries Containing Solid‐State or Liquid Electrolytes,” Nature Communications 13 (2022): 1153, 10.1038/s41467-022-28792-w. PMC8894478 35241673

[96] Y. Huang , B. Shao , Y. Wang , and F. Han , “Solid‐State Silicon Anode With Extremely High Initial Coulombic Efficiency,” Energy & Environmental Science 16 (2023): 1569–1580, 10.1039/D2EE04057C.

[97] A. Song , W. Zhang , H. Guo , et al., “A Review on the Features and Progress of Silicon Anodes‐Based Solid‐State Batteries,” Advanced Energy Materials 13 (2023): 2301464, 10.1002/aenm.202301464.

[98] C. Yuan , J. Wu , W. Zhang , Y. Liu , X. Wang , and X. Gao , “Deciphering the Interfacial Instability Electro‐Chemo‐Mechanical Mechanism of Si‐Based all‐Solid‐State Batteries: A Three‐Dimensional Multiphysics Coupled Approach,” Nano Energy 151 (2026): 111842, 10.1016/j.nanoen.2026.111842.

[99] H. Huo , M. Jiang , Y. Bai , et al., “Chemo‐Mechanical Failure Mechanisms of the Silicon Anode in Solid‐State Batteries,” Nature Materials 23 (2024): 543–551, 10.1038/s41563-023-01792-x. PMC10990934 38278984

[100] L. Sun , Y. Liu , L. Wang , and Z. Jin , “Advances and Future Prospects of Micro‐Silicon Anodes for High‐Energy‐Density Lithium‐Ion Batteries: A Comprehensive Review,” Advanced Functional Materials 34 (2024): 2403032, 10.1002/adfm.202403032.

[101] J. Yao , Z. Yu , J. Ma , et al., “Revealing Interfacial Failure Mechanism of Silicon Based all Solid State Batteries via Cryogenic Electron Microscopy,” Nature Communications 16 (2025): 9731, 10.1038/s41467-025-64697-0. PMC12586697 41188241

[102] W. Yan , Z. Mu , Z. Wang , et al., “Hard‐Carbon‐Stabilized Li–Si Anodes for High‐Performance All‐Solid‐State Li‐Ion Batteries,” Nature Energy 8 (2023): 800–813, 10.1038/s41560-023-01279-8.

[103] S. Poetke , F. Hippauf , A. Baasner , S. Dörfler , H. Althues , and S. Kaskel , “Nanostructured Si−C Composites as High‐Capacity Anode Material for all‐Solid‐State Lithium‐Ion Batteries,” Batteries & supercaps 4 (2021): 1323–1334, 10.1002/batt.202100055.

[104] F. Liang , L. Yang , R. Hu , B. Yuan , M. Zhu , and J. Liu , “Mechanisms, Development, and Applications of Silicon‐Based Anodes in Solid State Batteries,” Chemical Science 17 (2026): 738–771, 10.1039/D5SC08445H. PMC12703492 41403464

[105] T. K. Schwietert , V. A. Arszelewska , C. Wang , et al., “Clarifying the Relationship Between Redox Activity and Electrochemical Stability in Solid Electrolytes,” Nature Materials 19 (2020): 428–435, 10.1038/s41563-019-0576-0. 31932670

[106] Y. Zhu , X. He , and Y. Mo , “Origin of Outstanding Stability in the Lithium Solid Electrolyte Materials: Insights From Thermodynamic Analyses Based on First‐Principles Calculations,” ACS Applied Materials & Interfaces 7 (2015): 23685–23693, 10.1021/acsami.5b07517. 26440586

[107] Z. Cheng , M. Liu , S. Ganapathy , et al., “Revealing the Impact of Space‐Charge Layers on the Li‐Ion Transport in all‐Solid‐State Batteries,” Joule 4 (2020): 1311–1323, 10.1016/j.joule.2020.04.002.

[108] J. Haruyama , K. Sodeyama , L. Han , K. Takada , and Y. Tateyama , “Space–Charge Layer Effect at Interface Between Oxide Cathode and Sulfide Electrolyte in all‐Solid‐State Lithium‐Ion Battery,” Chemistry of Materials 26 (2014): 4248–4255, 10.1021/cm5016959.

[109] L. Wang , R. Xie , B. Chen , et al., “In‐Situ Visualization of the Space‐Charge‐Layer Effect on Interfacial Lithium‐Ion Transport in all‐Solid‐State Batteries,” Nature Communications 11 (2020): 5889, 10.1038/s41467-020-19726-5. PMC7674427 33208730

[110] D. Ren , L. Lu , R. Hua , et al., “Challenges and Opportunities of Practical Sulfide‐Based All‐Solid‐State Batteries,” eTransportation 18 (2023): 100272, 10.1016/j.etran.2023.100272.

[111] C. Zhu , S. Kobayashi , Y. Sugisawa , et al., “Space Charge Layer Evolution in all‐Solid‐State Batteries Probed via Operando Kelvin Probe Force Microscopy and Nuclear Reaction Analysis,” ACS Nano 19 (2025): 39062–39075, 10.1021/acsnano.5c10125. PMC12632181 41190736

[112] A. Sakuda , A. Hayashi , and M. Tatsumisago , “Interfacial Observation Between LiCoO_2_ Electrode and Li_2_S−P_2_S_5_ Solid Electrolytes of All‐Solid‐State Lithium Secondary Batteries Using Transmission Electron Microscopy,” Chemistry of Materials 22 (2010): 949–956, 10.1021/cm901819c.

[113] B. Gao , R. Jalem , and Y. Tateyama , “First‐Principles Study of Microscopic Electrochemistry at the LiCoO_2_ Cathode/LiNbO_3_ Coating/*β*‐Li_3_PS_4_ Solid Electrolyte Interfaces in an All‐Solid‐State Battery,” ACS Applied Materials & Interfaces 13 (2021): 11765–11773, 10.1021/acsami.0c19091. 33673737

[114] R. Koerver , I. Aygün , T. Leichtweiß , et al., “Capacity Fade in Solid‐State Batteries: Interphase Formation and Chemomechanical Processes in Nickel‐Rich Layered Oxide Cathodes and Lithium Thiophosphate Solid Electrolytes,” Chemistry of Materials 29 (2017): 5574–5582, 10.1021/acs.chemmater.7b00931.

[115] M. Ma , M. Zhang , B. Jiang , Y. Du , B. Hu , and C. Sun , “A Review of all‐Solid‐State Electrolytes for Lithium Batteries: High‐Voltage Cathode Materials, Solid‐State Electrolytes and Electrode–Electrolyte Interfaces,” Materials Chemistry Frontiers 7 (2023): 1268–1297, 10.1039/D2QM01071B.

[116] Q. Chen , Y. Pei , H. Chen , et al., “Highly Reversible Oxygen Redox in Layered Compounds Enabled by Surface Polyanions,” Nature Communications 11 (2020): 3411, 10.1038/s41467-020-17126-3. PMC7343805 32641725

[117] J. Seok , C. Chung , H. Lee , et al., “Decoupling Chemical and Mechanical Contributions to Capacity Fading in Ni‐Rich Cathodes for Sulfide‐Based all‐Solid‐State Batteries,” Advanced Energy Materials (2026): 06351, 10.1002/aenm.202506351.

[118] W. Kim , J. Noh , S. Lee , et al., “Aging Property of Halide Solid Electrolyte at the Cathode Interface,” Advanced Materials 35 (2023): 2301631, 10.1002/adma.202301631. 37122113

[119] T. Famprikis , P. Canepa , J. A. Dawson , M. S. Islam , and C. Masquelier , “Fundamentals of Inorganic Solid‐State Electrolytes for Batteries,” Nature Materials 18 (2019): 1278–1291, 10.1038/s41563-019-0431-3. 31427742

[120] F. Han , T. Gao , Y. Zhu , K. J. Gaskell , and C. Wang , “A Battery Made From a Single Material,” Advanced Materials 27 (2015): 3473–3483, 10.1002/adma.201500180. 25925023

[121] Z. Zhang , Y. Shao , B. Lotsch , et al., “New Horizons for Inorganic Solid State Ion Conductors,” Energy & Environmental Science 11 (2018): 1945–1976, 10.1039/C8EE01053F.

[122] M. Shoaib , P. Vallayil , N. Jaiswal , et al., “Advances in Redox Flow Batteries—A Comprehensive Review on Inorganic and Organic Electrolytes and Engineering Perspectives,” Advanced Energy Materials 14 (2024): 2400721, 10.1002/aenm.202400721.

[123] S. Wenzel , S. Randau , T. Leichtweiß , et al., “Direct Observation of the Interfacial Instability of the Fast Ionic Conductor Li_10_GeP_2_S_12_ at the Lithium Metal Anode,” Chemistry of Materials 28 (2016): 2400–2407, 10.1021/acs.chemmater.6b00610.

[124] J. Xu , T. Li , Y. Wang , et al., “Decoding NASICON and Its Metal Interface for Solid‐State Batteries,” Advanced Materials 38 (2026): 20270, 10.1002/adma.202520270. PMC13003917 41691429

[125] X.‐B. Cheng , C.‐Z. Zhao , Y.‐X. Yao , H. Liu , and Q. Zhang , “Recent Advances in Energy Chemistry Between Solid‐State Electrolyte and Safe Lithium‐Metal Anodes,” Chemistry 5 (2019): 74–96, 10.1016/j.chempr.2018.12.002.

[126] J. Huang , C. Li , D. Jiang , et al., “Solid‐State Electrolytes for Lithium Metal Batteries: State‐of‐the‐Art and Perspectives,” Advanced Functional Materials 35 (2025): 2411171, 10.1002/adfm.202411171.

[127] X. Zhang , H. Gao , and M. S. Wang , “Revealing the Failure Mechanisms of Lithium Metal Solid‐State Batteries With Solid Inorganic Electrolytes by in Situ Electron Microscopy,” Advanced Energy and Sustainability Research 6 (2024): 2400234, 10.1002/aesr.202400234.

[128] S. Choudhury , S. Stalin , D. Vu , et al., “Solid‐State Polymer Electrolytes for High‐Performance Lithium Metal Batteries,” Nature Communications 10 (2019): 4398, 10.1038/s41467-019-12423-y. PMC6765010 31562334

[129] F. Han , A. S. Westover , J. Yue , et al., “High Electronic Conductivity as the Origin of Lithium Dendrite Formation Within Solid Electrolytes,” Nature Energy 4 (2019): 187–196, 10.1038/s41560-018-0312-z.

[130] X. Xu , Y. Liu , O. O. Kapitanova , Z. Song , J. Sun , and S. Xiong , “Electro–Chemo–Mechanical Failure of Solid Electrolytes Induced by Growth of Internal Lithium Filaments,” Advanced Materials 34 (2022): 2207232, 10.1002/adma.202207232. 36148601

[131] R. Koerver , W. Zhang , L. de Biasi , et al., “Chemo‐Mechanical Expansion of Lithium Electrode Materials—on the Route to Mechanically Optimized All‐Solid‐State Batteries,” Energy & Environmental Science 11 (2018): 2142–2158, 10.1039/C8EE00907D.

[132] T. Krauskopf , F. H. Richter , W. G. Zeier , and J. Janek , “Physicochemical Concepts of the Lithium Metal Anode in Solid‐State Batteries,” Chemical Reviews 120 (2020): 7745–7794, 10.1021/acs.chemrev.0c00431. 32786669

[133] J. Lee , T. Lee , K. Char , K. J. Kim , and J. W. Choi , “Issues and Advances in Scaling up Sulfide‐Based All‐Solid‐State Batteries,” Accounts of Chemical Research 54 (2021): 3390–3402, 10.1021/acs.accounts.1c00333. 34402619

[134] C. Yuan , L. Qin , G. Xu , et al., “Operando Investigation of Sulfide‐Based all‐Solid‐State Lithium Batteries via Raman Spectroscopy: A Review,” Energy Storage Materials 76 (2025): 104110, 10.1016/j.ensm.2025.104110.

[135] C. Park , J. Choi , S. Park , et al., “Interfacial Chemistry‐Driven Reaction Dynamics and Resultant Microstructural Evolution in Lithium‐Based All‐Solid‐State Batteries,” Nature Communications 16 (2025): 8838, 10.1038/s41467-025-63959-1. PMC12494980 41044078

[136] X. Zheng , Z. Xue , H. Hao , et al., “Unravelling Electro‐Chemo‐Mechanical Interplay in Layered Oxide Cathode Degradation in Solid‐State Batteries,” Science Advances 11 (2025): ady7189, 10.1126/sciadv.ady7189. PMC12506959 41061061

[137] K. M. Kwon , M. Lee , D. H. Kim , H. R. Kang , and T. J. Park , “Minimum Effective Thickness of Cathode Protective Layers for Sulfide‐Based All‐Solid‐State Batteries via Powder‐Atomic Layer Deposition,” Energy Storage Materials 86 (2026): 105027, 10.1016/j.ensm.2026.105027.

[138] T. Huang , Y. Zheng , J. Ma , et al., “Cavity‐Engineered Polycrystalline Cathodes Resolve Stress Concentration Problem in all‐Solid‐State Lithium Metal Batteries,” Advanced Materials 38 (2026): 72940, 10.1002/adma.72940. 41888303

[139] J. Sang , B. Tang , K. Pan , Y.‐B. He , and Z. Zhou , “Current Status and Enhancement Strategies for all‐Solid‐State Lithium Batteries,” Accounts of Materials Research 4 (2023): 472–483, 10.1021/accountsmr.2c00229.

[140] R. Narayan , C. Laberty‐Robert , J. Pelta , J.‐M. Tarascon , and R. Dominko , “Self‐Healing: An Emerging Technology for Next‐Generation Smart Batteries,” Advanced Energy Materials 12 (2022): 2102652, 10.1002/aenm.202102652.

[141] W. Ma , S. Wan , X. Cui , et al., “Exploration and Application of Self‐Healing Strategies in Lithium Batteries,” Advanced Functional Materials 33 (2023): 2212821, 10.1002/adfm.202212821.

[142] Y. Wang , Y. Zhang , L. Su , et al., “Viscoelasticity and Self‐Healing Property of Dynamic Covalent Polymers: A Molecular Dynamics Simulation,” Polymer 295 (2024): 126775, 10.1016/j.polymer.2024.126775.

[143] J. Liu and M. W. Urban , “Dynamic Interfaces in Self‐Healable Polymers,” Langmuir 40 (2024): 7268–7285, 10.1021/acs.langmuir.3c03696. 38395626

[144] M. Si , X. Jian , Y. Xie , et al., “A Highly Damping, Crack‐Insensitive and Self‐Healable Binder for Lithium‐Sulfur Battery by Tailoring the Viscoelastic Behavior,” Advanced Energy Materials 14 (2024): 2303991, 10.1002/aenm.202303991.

[145] L. Wang , Z. Wang , Z. Su , B. Fang , and R. Mo , “Interface‐Engineered Strategy on Metal–Organic Framework to Chemical Stabilize PVDF‐HFP as Self‐Healing High‐Voltage Quasi‐Solid‐State Electrolyte,” Small 21 (2025): 08318, 10.1002/smll.202508318. 40817568

[146] Z. Yang , Y. Ye , N. Meng , and F. Lian , “Adaptive 3D Cross‐Linked Single‐Ion Conducting Polymer Electrolytes Enable Powerful Interface for Solid State Batteries,” Angewandte Chemie 137 (2025): 202505232, 10.1002/ange.202505232. 40728109

[147] R. Huang , R. Xu , J. Zhang , et al., “PVDF‐HFP‐Sn‐Based Gel Polymer Electrolyte for High‐Performance Lithium‐Ion Batteries,” Nano Research 16 (2023): 9480–9487, 10.1007/s12274-023-5707-x.

[148] Y. He , C. Wang , R. Lin , et al., “A Self‐Healing, Flowable, yet Solid Electrolyte Suppresses Li‐Metal Morphological Instabilities,” Advanced Materials 36 (2024): 2406315, 10.1002/adma.202406315. 39385628

[149] A. Marinow , Z. Katcharava , and W. H. Binder , “Self‐Healing Polymer Electrolytes for Next‐Generation Lithium Batteries,” Polymers 15 (2023): 1145, 10.3390/polym15051145. PMC10007462 36904385

[150] X. Gao , B. Liu , B. Hu , et al., “Solid‐State Lithium Battery Cathodes Operating at Low Pressures,” Joule 6 (2022): 636–646, 10.1016/j.joule.2022.02.008.

[151] Z. Huang , Z. Guo , H. Zhou , et al., “Liquid Metal‐Based Soft Materials for Self‐Healing Flexible Electronics,” Advanced Materials Technologies 11 (2026): 01524, 10.1002/admt.202501524.

[152] M. Song , B. Yu , W. Cui , W. Yang , Q. Bai , and Z. Zhang , “A Self‐Healing Room‐Temperature Liquid Eutectic Gasn Anode With Improved Wettability for Advanced Mg Ion Batteries,” Chemical Engineering Journal 435 (2022): 134903, 10.1016/j.cej.2022.134903.

[153] Y. Sun , Y. Wu , Y. Yan , et al., “Creep Localization Empowering High‐Capacity Alloy Anodes for Durable All‐Solid‐State Lithium Batteries,” Advanced Materials 37 (2025): 2510128, 10.1002/adma.202510128. 40662238

[154] Z. Geng , Y. Sun , Q. Zhang , et al., “An Elastomeric Lithium‐Conducting Interlayer for High‐Performance LATP‐Based Lithium Metal Batteries,” Small 20 (2024): 2402041, 10.1002/smll.202402041. 38855994

[155] J. Kim , J. Jung , J. Park , et al., “Self‐Healing Si Anodes With Robust Ionic and Electronic Conducting Network by Ga‐In‐Sn Liquid Metal Alloy in Solid‐State Batteries,” Energy Storage Materials 76 (2025): 104108, 10.1016/j.ensm.2025.104108.

[156] S. G. Yoon , B. S. Vishnugopi , D. L. Nelson , et al., “Interface Morphogenesis With a Deformable Secondary Phase in Solid‐State Lithium Batteries,” Science 388 (2025): 1062–1068, 10.1126/science.adt5229. 40472090

[157] H. Wan , C. Chen , N. Zhang , S. Huang , and X. Yao , “Potential‐Driven Selective Na Metal Deposition‐Enabled Interphase With High Lithium Dendrite Suppression Capability,” Journal of the American Chemical Society 147 (2025): 44852–44859, 10.1021/jacs.5c12341. 41269677

[158] Y. Cheng , M. Li , X. Yang , et al., “Na–K Alloy Anode for High‐Performance Solid‐State Sodium Metal Batteries,” Nano Letters 22 (2022): 9614–9620, 10.1021/acs.nanolett.2c03718. 36454039

[159] X. Guo , Y. Liu , X. Zhang , et al., “Revealing the Solid‐State Electrolyte Interfacial Stability Model With Na–K Liquid Alloy,” Angewandte Chemie International Edition 61 (2022): 202203409, 10.1002/anie.202203409. 35583391

[160] F. Pei , L. Wu , Y. Zhang , et al., “Interfacial Self‐Healing Polymer Electrolytes for Long‐Cycle Solid‐State Lithium‐Sulfur Batteries,” Nature Communications 15 (2024): 351, 10.1038/s41467-023-43467-w. PMC10774406 38191779

[161] Y. He , P. Zhao , L. Wang , Y. Jing , Y. Li , and H. L. Xin , “An Adhesive Adaptation Layer Mitigates the Interfacial Instabilities of Rigid Polymer Electrolyte,” Angewandte Chemie International Edition 64 (2025): 202424304, 10.1002/anie.202424304. 39956656

[162] X. Yang , Z. Zhang , X. Xu , et al., “Self‐Healing Functional Materials for Advanced Batteries: Mechanisms, Dynamics, and Applications,” Energy Storage Materials 75 (2025): 103992, 10.1016/j.ensm.2024.103992.

[163] H. Lv , X. Chu , Y. Zhang , Q. Liu , F. Wu , and D. Mu , “Self‐Healing Solid‐State Polymer Electrolytes for High‐Safety and Long‐Cycle Lithium‐Ion Batteries,” Materials Today 78 (2024): 181–208, 10.1016/j.mattod.2024.06.018.

[164] X. Cao , P. Zhang , N. Guo , et al., “Self‐Healing Solid Polymer Electrolyte Based on Imine Bonds for High Safety and Stable Lithium Metal Batteries,” RSC Advances 11 (2021): 2985–2994, 10.1039/D0RA10035H. PMC8694013 35424250

[165] S. Yang , S. Park , S. Kim , and S.‐K. Kim , “Vitrimer With Dynamic Imine Bonds as a Solid‐State Electrolyte for Lithium Metal Batteries,” Materials Today Energy 45 (2024): 101690, 10.1016/j.mtener.2024.101690.

[166] S. Cho , S. Y. Hwang , D. X. Oh , and J. Park , “Recent Progress in Self‐Healing Polymers and Hydrogels Based on Reversible Dynamic B–O Bonds: Boronic/Boronate Esters, Borax, and Benzoxaborole,” Journal of Materials Chemistry A 9 (2021): 14630–14655, 10.1039/D1TA02308J.

[167] Y. Wei , Y. Zhang , H. Wang , et al., “Self‐Healing Supramolecular Cathode Binder Additive With Dynamic Bonds for Durable High‐Voltage Solid‐State Lithium Batteries,” eScience 6 (2026): 100555, 10.1016/j.esci.2026.100555.

[168] Y. Zhao , T. Ma , L. Hu , X. Ren , X. Sun , and X. Yu , “Supramolecular Interaction Chemistry in Polymer Electrolytes towards Stable Lithium Metal Batteries,” Journal of Energy Chemistry 107 (2025): 154–169, 10.1016/j.jechem.2025.03.025.

[169] T. Sun , Q. Nian , X. Ren , and Z. Tao , “Hydrogen‐Bond Chemistry in Rechargeable Batteries,” Joule 7 (2023): 2700–2731, 10.1016/j.joule.2023.10.010.

[170] P. Zhang , Y. Liu , J. Wei , et al., “An Amphoteric and Hydrogen‐Bond‐Rich Artificial Α‐Amino Acid for Highly Durable Aqueous Redox Flow Batteries,” Nature Communications 16 (2025): 4727, 10.1038/s41467-025-59962-1. PMC12095679 40399283

[171] X. Lin , S. Xu , Y. Tong , et al., “A Self‐Healing Polymerized‐Ionic‐Liquid‐Based Polymer Electrolyte Enables a Long Lifespan and Dendrite‐Free Solid‐State Li Metal Batteries at Room Temperature,” Materials Horizons 10 (2023): 859–868, 10.1039/D2MH01289H. 36602156

[172] X. Hu , Z. Zhang , X. Zhang , et al., “External‐Pressure–Electrochemistry Coupling in Solid‐State Lithium Metal Batteries,” Nature Reviews Materials 9 (2024): 305–320, 10.1038/s41578-024-00669-y.

[173] L. Yang , S. Zhang , X. Liu , et al., “Multifunctional Polyurethane Phase‐Change Materials with Self‐Healing and Structural Integrity for Battery Thermal Management,” Chemical Engineering Journal 523 (2025): 168919, 10.1016/j.cej.2025.168919.

[174] J. Zhou , M. L. Holekevi Chandrappa , S. Tan , et al., “Healable and Conductive Sulfur Iodide for Solid‐State Li–S Batteries,” Nature 627 (2024): 301–305, 10.1038/s41586-024-07101-z. 38448596

[175] P. Barai , T. Fuchs , E. Trevisanello , F. H. Richter , J. Janek , and V. Srinivasan , “Study of Void Formation at the Lithium|Solid Electrolyte Interface,” Chemistry of Materials 36 (2024): 2245–2258, 10.1021/acs.chemmater.3c01708.

[176] K. Li , J. Huang , X. Qu , et al., “Void Evolution at the Li/LLZO Interface: Stack Pressure and Operating Temperature‐Driven Creep Effect,” ACS Applied Materials & Interfaces 17 (2025): 3146–3162, 10.1021/acsami.4c13564. 39760427

[177] S. Wang , L. Wen , X. Gong , J. Liang , X. Hou , and F. Hou , “Piezoelectric‐Based Energy Conversion and Storage Materials,” Batteries 9 (2023): 371, 10.3390/batteries9070371.

[178] D.‐C. Kong , Q.‐Y. Zhu , D.‐H. Guan , et al., “A Piezocatalysis Strategy to Enable Efficient Redox in Solid‐State Battery,” Angewandte Chemie International Edition 64 (2025): 202418174, 10.1002/anie.202418174. 39460426

[179] L. Wu , H. Lv , R. Zhang , et al., “Ferroelectric BaTiO_3_ Regulating the Local Electric Field for Interfacial Stability in Solid‐State Lithium Metal Batteries,” ACS Nano 18 (2024): 5498–5509, 10.1021/acsnano.3c10870. 38314720

[180] S.‐F. Li , M. Zuo , J.‐M. Wang , et al., “Macroscopically Ordered Piezo‐Potential in all‐Polymetric Solid Electrolytes Responding to Li Anode Volume Changes for Dendrites Suppression,” Advanced Science 13 (2025): 09897, 10.1002/advs.202509897. PMC12786380 41082343

[181] M. Kim , M. Kim , J. Jung , et al., “Suppression of Lithium Dendrite Growth Through Enhanced Ion Conductivity Using Lithium‐Doped ZnO in PVDF‐HFP Separators,” ECS Meeting Abstracts (2025): 410, 10.1149/MA2025-013410mtgabs.

[182] X. Wang , Y. Ying , Y. Gong , et al., “Piezoelectric Gradient Electrolytes for Environmentally Adaptive and Stable Zinc Batteries,” Energy Storage Materials 82 (2025): 104586, 10.1016/j.ensm.2025.104586.

[183] C.‐X. Zhao , Z. Li , B. Chen , F. Chen , and C. Wang , “Self‐Adaptive Electrolytes for Fast‐Charging Batteries,” Nature Energy 10 (2025): 904–913, 10.1038/s41560-025-01801-0.

[184] J. Fu , C. Wang , S. Wang , et al., “A Cost‐Effective All‐in‐One Halide Material for all‐Solid‐State Batteries,” Nature 643 (2025): 111–118, 10.1038/s41586-025-09153-1. 40562942

[185] G. Cen , H. Yu , R. Xiao , et al., “Adaptive Interphase Enabled Pressure‐Free All‐Solid‐State Lithium Metal Batteries,” Nature Sustainability 8 (2025): 1360–1370, 10.1038/s41893-025-01649-y.

[186] S. Lu , Y. Zhang , X. Zhang , et al., “Tailoring Argyrodite Electrolyte for Enhanced Interface Compatibility with Lithium Anode,” Chemical Engineering Journal 507 (2025): 160455, 10.1016/j.cej.2025.160455.

